# Emerging regulatory role of lncRNA MALAT1 in bone homeostasis: from the β–catenin–OPG/Jagged1 signalling axis to a broader regulatory network

**DOI:** 10.3389/fmed.2026.1980028

**Published:** 2026-10-23

**Authors:** Yangkai Shi, Zikang Xie

**Affiliations:** Department of Orthopedics-Traumatology V, Changzhou Hospital Affiliated to Nanjing University of Chinese Medicine, Changzhou, Jiangsu, China

**Keywords:** bone homeostasis, bone remodeling, Jagged1, MALAT1, OPG, osteoclast, osteogenic differentiation, β-catenin

## Abstract

In recent years, metastasis-associated lung adenocarcinoma transcript 1 (MALAT1) has emerged as a candidate regulator of bone homeostasis, with reported effects on the differentiation, activity and communication of osteoblasts and osteoclasts. These effects are cell-type specific, and in some experimental models they act in opposing directions. The remodeling of bone is a highly regulated process that is crucial to the integrity of the skeleton, and when misregulated, can cause metabolic bone disease, like osteoporosis and osteoarthritis. Studies in cultured cells and in rodent models indicate that MALAT1 acts on bone remodeling in part through the β-Catenin-OPG/Jagged1 pathway, affecting both osteogenic differentiation and osteoclastogenesis. In addition to this pathway, MALAT1 also regulates mesenchymal stem cell differentiation and the bone microenvironment, thus participating in a complex network of regulation that involves several signaling cascades and gene expression programs. Recent development of high throughput sequencing and functional assays has broadened the spectrum of MALAT1's role in bone metabolism, suggesting that it participates in several signalling axes rather than acting through a single dominant mechanism. This review aims to comprehensively summarize the molecular mechanisms by which MALAT1 regulates bone homeostasis, focusing on its interactions with the β-Catenin pathway and its influence on other regulatory pathways. To help clarify these pathways, we seek to advance understanding of the pathogenesis of bone metabolic disorders and to identify candidate targets for clinical intervention. Throughout, experimentally demonstrated mechanisms are distinguished from proposed models. The evidence base summarised here is almost entirely preclinical, and MALAT1 has not yet been validated as a biomarker or therapeutic target in any clinical study.

## 1 Introduction

Beyond their original characterization as “transcriptional noise,” long non-coding RNAs (lncRNAs) are now known to play key roles in regulation of gene expression and cellular processes. One of these is metastasis associated lung adenocarcinoma transcript 1 (MALAT1) or nuclear-enriched abundant transcript 2 (NEAT2), which is highly evolutionarily conserved and nuclear localized in mammalian species. MALAT1 was first recognised as a prognostic factor for metastasis in lung cancer (1, 2), and was later linked to breast, ovarian and hepatocellular carcinoma (3–5) and to non-malignant processes including sepsis and viral infection (6, 7). Its reported molecular activities include regulation of gene expression, alternative splicing, epigenetic regulation and binding to transcription factors and chromatin modifiers (1, 8, 9). These functions indicate that MALAT1 contributes to nuclear architecture and to gene regulatory networks, although the extent to which it occupies a hierarchically dominant position within those networks has not been established.

Although the role of MALAT1 in oncogenesis has been described in detail, recent studies have uncovered its essential role in tissue homeostasis and development. Remarkably, MALAT1 is involved in the regulation of cell proliferation, migration, apoptosis and inflammatory responses in various microenvironments, such as the ovarian follicle and the feto-maternal interface, pointing to its role in reproductive biology and related disorders. The findings point to the versatility of MALAT1 in controlling the cellular phenotypes in response to physiological and pathological stimuli.

At the same time, the skeletal system is a dynamic system where the exact regulation of the bone remodeling process is a critical determinant of bone homeostasis has been garnering more attention. Bone remodeling is a well-regulated process involving the coordinated activity of bone-forming cells (osteoblasts) and bone-resorbing cells (osteoclasts). Any disturbance of this balance leads to disturbance of the skeleton such as osteoporosis or osteopetrosis, making knowledge of the molecular regulators of bone cell differentiation and function important. MALAT1 is one of the emerging lncRNAs which are key parts of the system affecting bone remodeling (8).

Bone remodeling proceeds as a cycle within the basic multicellular unit, in which a period of osteoclastic resorption is followed by a reversal phase and then by osteoblastic formation, so that under normal conditions the amount of bone removed is matched by the amount replaced (10). Osteocytes, the most abundant cell in bone, act as the principal mechanosensors of this cycle and regulate both of its arms, in part by secreting sclerostin to restrain Wnt signalling in osteoblasts and by releasing RANKL that supports osteoclast formation (11). The central coupling module is the RANK-RANKL-OPG axis: osteoblast lineage cells present RANKL to RANK on osteoclast precursors and thereby drive their differentiation, while osteoprotegerin secreted by the same lineage acts as a decoy receptor that neutralises RANKL, so that it is the ratio of RANKL to OPG rather than the level of either molecule alone that determines resorptive activity (12). Mechanical loading, parathyroid hormone, oestrogen and inflammatory cytokines all converge on this ratio. The contribution of MALAT1 described below should be read against this background, because most of the reported effects act on one of these established coupling steps rather than through a separate pathway.

Importantly, MALAT1 has been shown to regulate several signaling pathways that are known to play a role in osteogenesis and osteoclastogenesis, which make its role in bone homeostasis particularly intriguing. Among these, a major signal transduction pathway is the so called Wnt/β-Cateninsignaling pathway which is involved in the control of osteoblast differentiation and activity. The regulatory role of MALAT1 in this pathway has been uncovered and downstream targets such as osteoprotegerin (OPG) and Jagged1 which are key factors in bone metabolism are known to be targeted by MALAT1. OPG inhibits the differentiation and activity of osteoclasts as well as through its interaction with Jagged1, which regulates the fate of bone progenitor cells, by acting as a decoy receptor for receptor activator of nuclear factor kappa-Β ligand (RANKL). These molecules together form a regulatory axis through which MALAT1 influences bone remodeling. The direction of that influence is not uniform: MALAT1 has been reported to favour osteogenic differentiation in osteoblast and mesenchymal stem cell lineages, yet to favour osteoclastogenesis in monocyte-macrophage lineage cells, so its net effect on bone mass is context dependent rather than simply negative (13–15).

Besides the β-Catenin-OPG/Jagged1 pathway, MALAT1 can regulate cellular components in the bone microenvironment by competitive endogenous RNA (ceRNA), epigenetic modification, as well as targeting different transcription factors. They involve bone marrow mesenchymal stem cells (BMSCs), chondrocytes and immune cells, all of which are involved in the dynamic equilibrium of bone formation and resorption. Another form of post-transcriptional regulation involved in bone cell function is the MALAT1 ceRNA mechanism whereby the binding of microRNAs sequesters them and thus fine-tunes gene expression (8).

There are emerging evidences of the role of MALAT1 in bone diseases. The deregulation of MALAT1 expression has been associated with changes in bone remodeling which play a role in pathogenesis of osteoporosis and other metabolic bone disorders. The molecular mechanisms through which MALAT1 acts in bone cells suggest candidate drug development targets, and restoration of MALAT1 expression has improved bone phenotypes in preclinical models of osteoporosis (14, 16).

Given this context, it is opportune to review the existing knowledge on the myriad of functions of MALAT1 in bone biology. The aim of this review is to systematically elaborate MALAT1's role in bone homeostasis, beginning with its role in the regulation of the β-Catenin-OPG/Jagged1 pathway and then moving on to the wider bone microenvironment. Besides, the function of MALAT1 dysregulation in bone diseases and the possible therapeutic potential of MALAT1 in bone diseases will be explained and understood. This integrative perspective is intended to clarify the regulatory role that MALAT1 appears to occupy in bone remodeling, and to show how far the present evidence extends towards application in skeletal medicine.

## 2 Molecular characteristics of MALAT1 and the biological basis of bone homeostasis

### 2.1 Gene structure, expression, and subcellular localization of MALAT1

Metastasis-associated lung adenocarcinoma transcript 1 (MALAT1) (also termed nuclear-enriched abundant transcript 2, NEAT2) is a long non-coding RNA (lncRNA) that has gained considerable interest due to its highly conserved gene structure, expression pattern and unique subcellular localization, which form the fundamental attributes of the gene that underlie its regulatory functions in various physiological and pathological processes. MALAT1 is a gene that resides on human chromosome 11q13 and has a length of about 8.7 kilobases (kb). This is a region that contains a very conserved nuclear retention signal essential for its nuclear localization and activity (5). MALAT1 is highly conserved among mammals, indicating a conserved function (8).

The subcellular localization of MALAT1 is mainly in nuclear speckles, which are dynamic subnuclear structures that are associated with pre-mRNA splicing factors, and are involved in gene expression and RNA processing regulation. The conserved nuclear retention elements contribute to the nuclear localization and enable MALAT1 to be utilized as a scaffold and/or regulator within nuclear compartments (9). Due to its nuclear localization, MALAT1 is able to directly influence post-transcriptional and transcriptional mechanisms including alternative splicing and mRNA maturation by interacting with a variety of RNA binding proteins and splicing machinery components (1). This spatial localization is very important for the role of MALAT1 as a regulator of expression of important programs essential for maintenance of cell homeostasis.

Multiple transcription factors and epigenetic modifications are involved in the regulation of MALAT1 expression. There are several SP1/3 binding sites in the MALAT1 promoter region, and specificity protein 1 (SP1) and specificity protein 3 (SP3) have been found as important upstream regulators that bind to the MALAT1 promoter region. Experimental investigation in hepatocellular carcinoma (HCC) cells revealed that SP1 and SP3 have a synergistic effect on the activation of expression of MALAT1, and that mithramycin A, an inhibitor of their binding to DNA, significantly reduces the expression of MALAT1, suggesting their role in MALAT1 expression regulation (5). Besides, the transcription of MALAT1 was found to be regulated by epigenetic mechanisms, such as histone acetylation, which has been found to regulate the expression of this gene, but the specific epigenetic marks remain to be clarified. These regulatory layers help to ensure that MALAT1 is expressed in a way that is sensitive to the cell context and environmental cues.

From the viewpoint of tissue expression, MALAT1 is ubiquitous and is among the most abundant long non-coding RNAs in most tissues examined. Reports that it is highly expressed in osteocytes, mesenchymal stem cells and osteoblasts should be read with two qualifications. The abundance of MALAT1 in bone lineage cells has been established mainly by RNA sequencing and quantitative PCR of cultured cells and of whole bone, not by comparison against a matched panel of other tissues, so it is abundance rather than bone enrichment that the data support. The functional studies that follow rely on changes in MALAT1 level during differentiation, which is a more informative measure than the absolute level; MALAT1 rises during osteogenic induction of mesenchymal stem cells and falls during osteoclastic differentiation of macrophages in the studies cited below (14, 17, 18). Because of its high expression in these cells, MALAT1 might play a role in the regulation of osteogenic differentiation and in bone remodeling events, perhaps in a regulatory fashion on gene expression networks related to bone metabolism (8).

MALAT1 localizes to the nucleus where it alters the function of splicing factors and chromatin modifiers to influence alternative splicing and transcription of genes. MALAT1 binds to the serine/arginine-rich splicing factors (SRSFs) and other nuclear proteins to regulate the splicing of pre-mRNAs, which may regulate the gene expression of downstream targets related to cell proliferation, differentiation, and migration (1). Bone homeostasis and correct gene expression and splicing of genes are critical to maintaining the balance between bone formation and bone resorption. In addition, MALAT1 has been shown to play a role in epigenetic control by remodeling the chromatin, further emphasizing its pleiotropic role in programs of gene expression that are relevant to bone physiology (19).

To summarize, MALAT1 is a highly conserved lncRNA that is approximately 8.7 kb in length, is encoded on chromosome 11q13, and contains nuclear retention sequences that restrict its localization to mainly nuclear speckles. It is regulated by transcription factors like SP1, SP3, and is modulated by epigenetic factors like histone acetylation. MALAT1 is highly expressed in bone-related cells and is localized in the nucleus allowing for direct regulation of gene transcription, alternative splicing and mRNA processing. All these properties make MALAT1 a crucial regulator of bone homeostasis by controlling the expression of bone remodeling and osteogenesis-related genes.

### 2.2 Overview of cellular and molecular regulatory networks in bone homeostasis

In order to maintain bone homeostasis, three cell types are involved in a coordinated activity: osteoblasts, osteoclasts and osteocytes. Bone cells are osteoblasts (bone forming cells), osteoclasts (bone resorbing cells) and osteocytes (bone mechanosensors and regulators). The Wnt/β-Catenin, RANKL/OPG and Notch pathways are the most prominent of the many signaling pathways that balance bone formation and resorption.

The Wnt/β-Catenin pathway is a key pathway in the differentiation of osteoblasts and their function. This pathway is activated to stimulate osteogenic gene expression to stimulate bone formation. Rightside, on the other hand, disorders can result in poor osteogenesis and bone disorders. Two important modulators of osteoclastogenesis produced by osteoblasts are osteoprotegerin (OPG) and receptor activator of nuclear factor κB ligand (RANKL). RANKL is a factor that acts on the receptor RANK that is found on osteoclast precursor cells, which causes their maturation and activation and bone resorption. OPG competes with RANKL for binding with RANK, which prevents osteoclast formation. The balance of production of RANKL vs. OPG by the osteoblasts is important for bone remodeling balance (20).

The third pathway is Notch signalling, activated when a ligand of the Jagged or Delta-like family engages one of the four Notch receptors on an adjacent cell. Its skeletal effect is not uniform and cannot be reduced to a single direction. The outcome depends on which ligand is presented, which receptor is engaged and the differentiation stage of the responding cell: in early mesenchymal progenitors Notch activation maintains the progenitor pool and suppresses osteoblast differentiation, whereas in committed osteoblast lineage cells it supports matrix production, and in osteoclast precursors individual receptors have been reported to act in opposing directions (21). Notch and Wnt/β-Catenin signalling intersect at several points in osteoblast lineage cells, so the two are better described as mutually modifying than as acting in parallel (21, 22).

Newly identified, MALAT1 is a long non-coding RNA (lncRNA), which through a complex regulatory network, integrates these signaling pathways, and is essential for proper bone remodeling. MALAT1 is a molecular sponge regulating the differentiation of osteoblasts and osteoclasts binding to multiple microRNAs that regulate key signaling pathways. For instance, MALAT1 sponges miR-124-3p and thereby up-regulates IGF2BP1, which activates the Wnt/β-Catenin pathway; in that study MALAT1 promoted the osteogenic differentiation of bone marrow stem cells and reduced the osteoclastic differentiation of macrophages (14). Similarly, MALAT1 controls osteoclast differentiation through the miR-124/NFATc1 axis, and induces osteoclastogenesis in specific situations (15).

Furthermore, MALAT1 regulates the expression of OPG and RANKL in osteoblasts, thus indirectly regulating the activity of osteoclasts and the process of bone resorption. It was found that when the osteoblast was stimulated with RANKL, its expression of MALAT1 was known to regulate the expression level of OPG, thereby creating a feedback mechanism between RANKL/OPG axis and MALAT1 (20). In addition, MALAT1 can also interact with the Notch signaling pathway by Jagged1, promoting osteoblast differentiation and inhibiting the formation of osteoclasts which is consistent with MALAT1 acting as one of several regulatory components of the signals involved in bone remodeling (22).

MALAT1 is also regulated by other microRNAs and signaling molecules, adding to the complexity of the network. For example, MALAT1 is able to bind the microRNA miR-96, which functions as a regulator of the transcription factor Osterix (Osx), involved in osteogenesis, and thereby promotes osteogenesis (18). It also serves to control the expression of miR-129-5p, in order to guarantee the differentiation potential of osteoblasts derived from aged bone marrow stem cells (23). The different layers of interactions place MALAT1 in a central position, as an important modulator of the molecular signals regulating bone homeostasis.

The bone homeostasis is controlled by the activities of osteocytes, osteoblasts and osteoclasts and some of the most critical pathways that are involved are Wnt/β-Catenin, RANKL/OPG and Notch. MALAT1 intersects with these pathways as an important regulatory component, acting on microRNA activity and on gene expression to influence the balance of bone remodelling. Direct evidence placing MALAT1 hierarchically upstream of the Wnt/β-Catenin, RANKL/OPG and Notch pathways has not been reported, and the term upstream regulator is therefore not used. Knowledge of this complex network may give us a therapeutic opportunity for treatment of bone metabolism diseases such as osteoporosis and bone healing (14, 15, 18, 20, 22, 23).

## 3 MALAT1 regulates bone remodeling via the β-catenin-OPG/Jagged1 signaling axis

### 3.1 MALAT1 directly regulates the β-catenin signaling pathway

In addition, the expression of other pathways, such as the β-catenin signaling pathway, which is closely involved in osteogenesis and bone remodeling, was directly affected by another pathway, metastasis associated lung adenocarcinoma transcript 1 (MALAT1), which was also found to play a crucial role in maintaining bone homeostasis. The proposed mechanism is that MALAT1 associates with β-catenin and favours its nuclear translocation and transcriptional activity. A direct physical interaction between MALAT1 and the β-catenin protein has been inferred from co-precipitation and protein stabilisation experiments rather than demonstrated structurally, and it is therefore presented here as a proposed rather than an established mechanism. This interaction leads to the expression of target genes downstream such as Jagged1 and osteoprotegerin (OPG) proteins, which play a role in osteoblast–osteoclast interaction. The mouse evidence comes from two complementary models. Global Malat1 knockout mice on a C57BL/6 background were compared with wild-type littermates at 12 weeks of age in both sexes, and micro-computed tomography of the distal femur showed reduced trabecular bone volume fraction, trabecular number, bone mineral density and connectivity density with increased trabecular separation, while cortical bone was unaffected; static histomorphometry after TRAP staining showed increased osteoclast surface per bone surface and osteoclast number per bone perimeter, and calcein double labelling of the tibia showed a reduced mineral apposition rate and bone formation rate per bone surface. To establish that the defect originates in the osteoblast, Malat1 conditional knockout mice were generated by crossing Malat1 floxed animals with an osteocalcin promoter driven Cre line, and these osteoblast-specific animals reproduced the low bone mass phenotype (13). After the MALAT1 knockdown in osteoblast precursor cells, it was observed that the nuclear accumulation of β-catenin was significantly decreased and this was associated with a decrease in the activity of Wnt/β-catenin signaling. This downregulation coincided with the downregulation of transcription factors that are key for the maturation and activity of osteoblasts, such as Runx2 and Osterix, markers of osteogenic differentiation (24). The mechanism for the stabilizing effect of MALAT1 on β-catenin has been suggested to be through binding to the N-terminal domain of β-catenin to block its degradation by ubiquitination via the proteasome. This stabilization results in high levels of β-catenin protein, which helps to keep Wnt signaling active, which is important for bone formation (13).

In addition, the expression of MALAT1 has been shown to promote osteogenic differentiation via regulation of the miR-124-3p/IGF2BP1/Wnt/β-catenin pathway. The MALAT1 will bind with miR-124-3p, which will be a competing endogenous RNA (ceRNA), to block the miR-124-3p-mediated negative regulation of IGF2BP1, a positive regulator of β-catenin signaling. Upregulation of MALAT1 then, in turn, stimulates the activation of the Wnt/β-catenin pathway, which promotes osteoblast differentiation and may indirectly influence osteoclastogenesis through osteoblast-mediated coupling mechanisms, and thereby contributes to bone mass accumulation and maintenance (14). This ceRNA mechanism shows how the MALAT1 has several regulatory functions that act on the β-catenin signaling pathway both at the transcriptional and post transverse level.

MALAT1 is also involved in the regulation of other cells involved in bone remodeling, such as chondrocytes. It regulates cellular crosstalk through the regulation of activation of the β-catenin/OPG/Jagged1 pathway, which is an important pathway responsible for integrating signals and coordinating bone formation and bone resorption. MALAT1 is not a direct inhibitor of osteoclastogenesis, but instead regulates osteoblasts and chondrocytes, which in turn can affect osteoclastogenesis, in a non-cell autonomous fashion (13). Moreover, this regulatory network is also engaged in pathologies such as fracture healing, where MALAT1 is highly expressed, leading to a faster healing process via activation of the β-catenin signaling pathway and its effectors.

These findings have been corroborated by other studies in other cell types and disease models that suggest a role for MALAT1 in β-catenin signaling. It has been shown that metabolites of L. fermentum GBE18 induce the expression of MALAT1 in pre-osteoblasts (MC3T3-E1), which results in the stabilization of β-catenin and the increase of Runx2, and then further promote the osteogenic differentiation of pre-osteoblasts in a coordinated manner by activating the Wnt/β-catenin and PI3K/Akt pathways (25). In contrast, the β-catenin signaling pathway is involved in tumor progression in cancer models, revealing the context-dependent functions of MALAT1 and its ability to function as a molecular regulator in various contexts (26).

These results provide a detailed understanding of the direct association of MALAT1 with β-catenin, which promotes the intranuclear localization and activation of MALAT1 and its ability to regulate the expression of other important genes involved in osteogenic function including OPG and Jagged1. This axis is a crucial factor in the differentiation of osteoblasts, formation of bone tissue and maintaining the integrity of the skeleton. MALAT1 is an important regulatory component of bone homeostasis, and disruption of its expression or activity affects β-catenin signaling, with reduced expression of osteogenic markers and loss of bone mass in the models examined. The main signaling pathway directly targeted by MALAT1 which stabilizes β-catenin and activates the expression of OPG and Jagged1 is indicated in Figure 1. In this model, MALAT1 acts on both bone formation and bone resorption signals through a shared molecular node. The model is a synthesis of separate experiments rather than a pathway that has been demonstrated end to end in a single experimental system.

**Figure 1:**
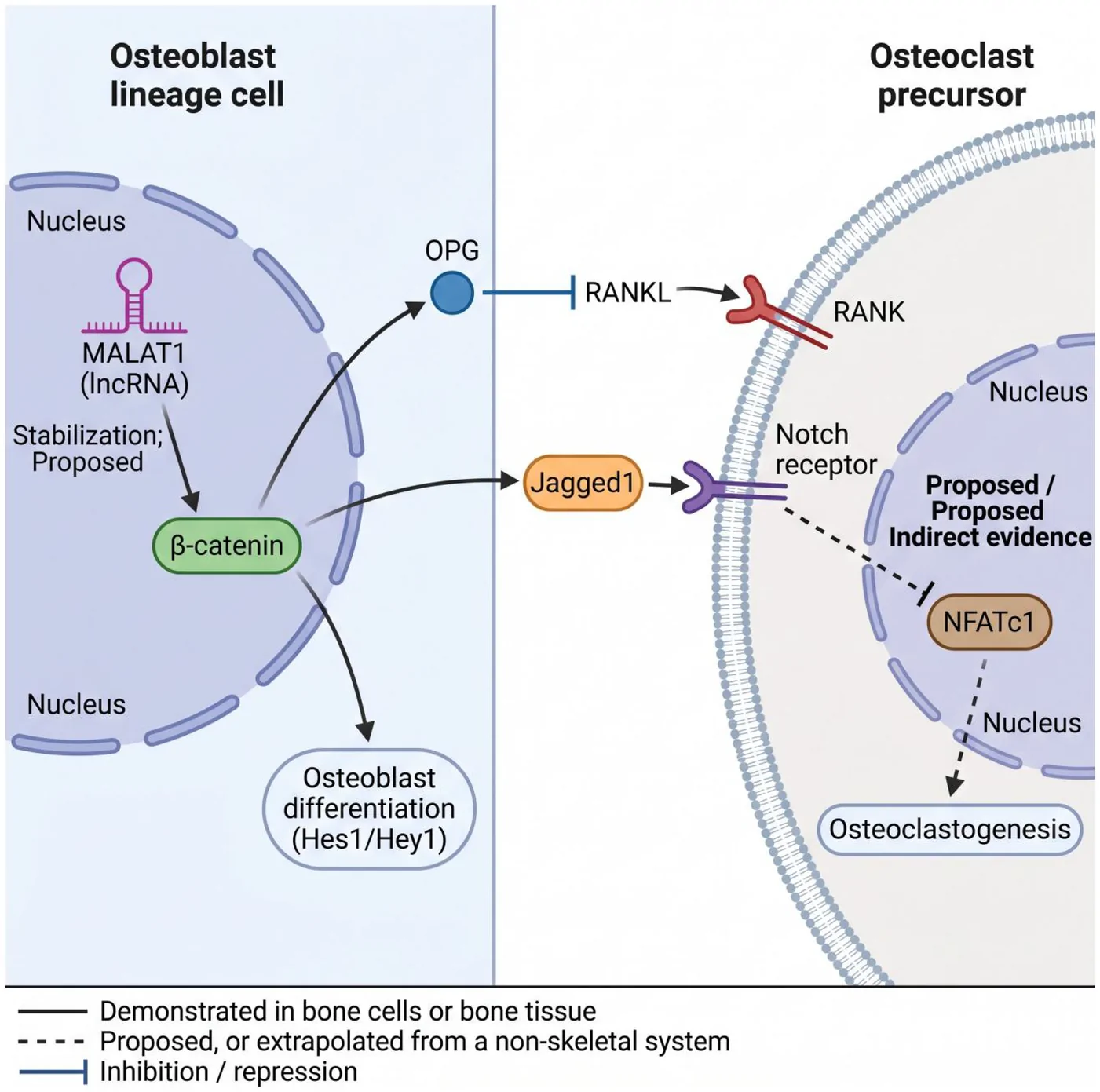
MALAT1 regulates bone homeostasis via the β-catenin-OPG/Jagged1 signaling axis. MALAT1 in the osteoblast lineage is proposed to stabilise β-catenin, favouring its nuclear translocation (this stabilisation is inferred from co-precipitation and protein stabilisation experiments, without structural demonstration). Nuclear β-catenin up-regulates two downstream targets, OPG and Jagged1. OPG acts as a decoy receptor for RANKL, blocking its binding to RANK on osteoclast precursors and thereby inhibiting osteoclastogenesis. Jagged1 activates Notch signalling in neighbouring osteoblasts to drive osteogenic maturation via Hes1/Hey1. The proposed inhibition of NFATc1 in osteoclast precursors by Notch activation is drawn as a dashed connector, as it rests on indirect evidence and was not demonstrated in the MALAT1 context. In Malat1 knockout mice, the increased resorptive phenotype is non-cell-autonomous and mediated by the osteoblast RANKL:OPG ratio rather than by a direct effect on osteoclasts. Solid connectors denote interactions demonstrated in bone cells or bone tissue; dashed connectors denote interactions that are proposed or extrapolated from non-skeletal systems; blunt-ended connectors denote inhibition. The figure is a synthesis across studies rather than a pathway shown end to end in a single experimental system.

### 3.2 OPG as a downstream effector of MALAT1 in inhibiting bone resorption

Osteoprotegerin (OPG) is an important endogenous inhibitor of receptor activator of nuclear factor kappa-Β ligand (RANKL) which is a potent regulator of osteoclast differentiation and activation. By blocking RANKL, OPG prevents its binding with the receptor activator of nuclear factor kappa-Β (RANK) on osteoclast precursors, thus blocking osteoclastogenesis and bone resorption. MALAT1 has been reported to increase OPG expression, an effect attributed to its action on β-catenin signalling. The proposed mechanism is that MALAT1 associates with β-catenin, favours its nuclear translocation and thereby raises transcription of the OPG gene. As noted in Section 3.1, the interaction with the β-catenin protein is inferred rather than demonstrated structurally, and the same qualification applies here. The *in vivo* studies also corroborate the role of MALAT1-OPG axis in bone homeostasis (13).

In mice, increased Malat1 expression was accompanied by higher serum OPG and lower serum C-terminal telopeptide of type 1 collagen (CTX-1). These are murine measurements obtained in genetically modified animals; no corresponding serum OPG or CTX-1 data from human subjects with altered MALAT1 expression have been reported, and the finding should not be generalised to patients. It is associated with a significant increase of the bone mineral density (BMD) and this indicates the physiological relevance of the upregulation of OPG expression by MALAT1 and its importance to maintain skeletal integrity. This pathway is involved in inhibiting the activity of osteoclasts, which suggests that MALAT1 may not only regulate bone remodeling via osteoblasts and chondrocytes, but also act indirectly to regulate osteoclastogenesis (13).

The available human data are narrower than a correlation between MALAT1 and OPG in bone tissue. What has been reported is that MALAT1 is lower in peripheral blood from elderly patients with osteoporosis than in healthy volunteers, and that MALAT1 falls in bone marrow stem cells during osteogenic differentiation *in vitro*. We are not aware of a study measuring MALAT1 and OPG together in human bone samples, so the link between the two in patients remains inferred from the mouse and cell work described above rather than demonstrated. This is the reason that the MALAT1-OPG signaling pathway has been thought to be a new important target for osteoporosis therapy, particularly humans (14).

MALAT1-β-catenin-OPG axis is a complex regulatory network, in which MALAT1 is a scaffold/molecular decoy, and participates in the stabilization of β-catenin and its transcription activities at the molecular level. This interaction leads to increased transcription of OPG, and affects the transcription of other target genes of β-catenin which are related to osteogenesis and bone remodeling. MALAT1 can act as both a positive and negative regulator of osteoblast differentiation and osteoclastogenesis, respectively, in the process of induction of OPG expression, making it an important regulator of bone homeostasis. Moreover, MALAT1 also croslinks between osteoblasts, osteoclasts and chondrocytes to reveal the complexity of the cell-cell interaction that is essential for the maintenance and repair of the skeleton (13).

Finally, bone resorption is an important downstream target of MALAT1 for its role in bone resorption inhibition in OPG. The MALAT1 interaction with β-catenin increases the binding of β-catenin to enhance the upregulation of OPG, which regulates osteoclast differentiation and activity. Changes in serum OPG accompanied by reduced bone resorption markers were observed in mice in which Malat1 was overexpressed (13). No equivalent measurements have been reported in patients, and the preceding sentence should not be read as implying a clinical correlation. The MALAT1-OPG axis is, therefore, an important bone remodeling regulatory pathway that might serve as a therapeutic target for bone metabolic diseases.

### 3.3 Function of Jagged1 in MALAT1-mediated osteoblast-osteoclast coupling regulation

The long non-coding RNA (lncRNA) MALAT1 is a key player in the complex regulation of bone remodeling, especially in the communication between osteoblasts and osteoclasts that is mediated by Jagged1, a canonical ligand of the Notch signaling pathway. In osteoblast lineage cells, MALAT1 is shown to activate the β-catenin signaling pathway, leading to the upregulation of Jagged1 transcription and activation of downstream Notch signaling pathway, which result in the differentiation of osteoblasts. Specifically, MALAT1 binds to β-catenin and promotes its nuclear retention and transcription activity, thereby making an increase in the expression of Jagged1 on the surface of the osteoblast. On the elevated surfaces, the Jagged1 is expressed and is responsible for binding to Notch receptors on neighboring osteoblasts, which activates the expression of Notch target genes that are important for osteogenic differentiation and maturation such as the genes coding for Hes1 and Hey1. This mechanism suggests that MALAT1 is an endogenous regulatory factor of osteogenesis which acts on the Jagged1-Notch-β-catenin axis (13).

It has been proposed that Jagged1 also acts on osteoclast precursors, where Notch activation would reduce induction of nuclear factor of activated T cells c1 (NFATc1), the transcription factor required for osteoclast differentiation. We were unable to identify a study that demonstrates this step for Jagged1 in the MALAT1 context, and the knockout work discussed above attributes the reduction in resorption to the RANKL to OPG ratio presented by osteoblasts rather than to a direct action on the precursor. This arm is therefore presented as a proposed mechanism, and it is drawn as such in Figure 1. Therefore, Jagged1 is an important molecule which can regulate the balance between bone formation and bone resorption by inducing osteoblast differentiation simultaneously inhibiting osteoclastogenesis, thus preserving bone homeostasis (13).

The two lineages do not respond to Jagged1 in the same way, and that difference matters for the axis described here. In osteoblast lineage cells, Jagged1 presented on a neighbouring cell engages Notch receptors and induces the HES and HEY transcriptional repressors, but the consequence depends on the stage of the responding cell: in uncommitted mesenchymal progenitors Notch activation restrains osteoblast differentiation and preserves the progenitor pool, whereas in cells already committed to the osteoblast lineage the same ligand supports matrix production (21). In osteoclast precursors the receptor complement is different, and individual Notch receptors have been reported to act in opposite directions on differentiation, so the effect of a given ligand in that lineage cannot be predicted from the osteoblast result (21). This is the reason the MALAT1-dependent Jagged1 arm should not be read as a single directional input acting identically on both lineages, and the reason the corresponding connector is drawn as proposed rather than demonstrated in Figure 1.

The mouse work supports a role for Jagged1 downstream of Malat1. Malat1 deficient mice show reduced Jagged1 in bone together with reduced bone formation, increased osteoclast number and surface, and deteriorated trabecular microarchitecture. Jagged1 is therefore best described as one of the mediators through which Malat1 acts on osteoblast and osteoclast coupling, alongside osteoprotegerin, rather than as the sole route (13).

Furthermore, numerous pathways of cellular crosstalk in the bone microenvironment are associated with MALAT1-β-catenin-Jagged1-Notch signaling axis. In addition to its role in the regulation of osteoblast lineage cells, MALAT1 regulates other cells, such as chondrocytes and osteoclasts indirectly, leading to complex regulation to control the precise process of bone remodeling and repair. Bones need to be both remodeled and remodeled again—Jagged1-Notch signaling in osteoblasts can turn up osteogenic gene expression while blocking the signaling in osteoclast precursors can reduce bone resorpion. Jagged1 therefore appears to be an important mediator of MALAT1-associated osteoblast and osteoclast coupling (13).

In summary, Jagged1 acts downstream of β-catenin signalling and induces the Notch target genes that support osteoblast differentiation. The further step, in which Jagged1 would reduce NFATc1 in osteoclast precursors, is proposed rather than demonstrated, as set out above, and should be read with that qualification here as well. These pathways are affected in MALAT1 deficient models, resulting in decreased bone formation, increased bone resorption and impaired bone microstructure, suggesting the significance of Jagged1 in bone health. The information gained helps to develop a mechanism that can serve as a basis for possible therapeutic options for developing bone metabolic diseases (BMD), like osteoporosis, by targeting MALAT1-Jagged1-Notch axis.

## 4 MALAT1 regulates bone-related gene expression through the ceRNA mechanism

### 4.1 MALAT1 as a miRNA sponge regulating key osteogenic transcription factors

MALAT1 has been reported to act on osteogenic transcription factors indirectly, through competing endogenous RNA interactions with microRNAs that repress them. The factors for which this has been tested in skeletal or dental cells are Runx2 (17), Osterix (18) and SMAD5 (22). In this ceRNA mechanism, the MALAT1 could suppress the inhibitory effects of some miRNAs on these transcription factors, which would contribute to osteoblast differentiation and bone formation, besides of the impacts on the β-Catenin pathway, and would be added to the bone homeostasis regulating mechanisms.

The mechanism of action of MALAT1 in its osteogenic effect is by competitive binding of miRNAs that inhibit osteogenic effects on key transcription factors that regulate osteoblast differentiation. The members of miR-30 family, such as miR-30, are target genes of MALAT1, which directly targets the master gene of osteogenesis, Runx2. In Adipose-Derived Mesenchymal Stem Cells (ADSCs), miR-30 expression is decreased while MALAT1 expression is increased during osteogenic induction. Reduced MALAT1 levels or increased miR-30 levels can lead to a decrease in Runx2 levels and downstream decrease in levels of osteogenic markers such as osteocalcin (OCN), osteopontin (OPN) and Osterix, which in turn leads to reduced mineralized nodule formation and impaired alkaline phosphatase (ALP) activity. MALAT1 overexpression on the contrary, allows the MALAT1 induced osteogenic differentiation through the interaction with miR-30 that is responsible for the suppression of the mRNA translation of RUNX2 (17, 27).

Similarly, MALAT1 also functions as a sponge of miR-96 to control the expression of another key osteoblast differentiation transcription factor, Osterix. The expression of MALAT1 is increased during osteogenic differentiation of human bone marrow-derived mesenchymal stem cells (hBMSCs), and that of miR-96 is decreased. Functional assays demonstrate that MALAT1 silencing decreases osteogenesis while the overexpression of miR-96 has a negative effect on the expression of Osterix and osteogenic markers. The direct binding of MALAT1 to miR-96 and the direct targeting of Osterix by miR-96 are confirmed by the dual-luciferase reporter assays which create a MALAT1/miR-96/Osterix axis that promotes the differentiation of osteoblasts (18).

Two further miRNAs have been linked to MALAT1 in osteogenic contexts, and the evidence for each is narrower than a combined statement would suggest. The MALAT1/miR-214 axis has been demonstrated in human bone marrow stromal cells, but its downstream effector is ATF4 rather than Runx2, and the setting is steroid-induced avascular necrosis of the femoral head (28). The MALAT1/miR-204 axis has been shown to promote osteoblastic differentiation through Smad4, but in human aortic valve interstitial cells rather than in skeletal cells, so its applicability to bone is an extrapolation (29). Neither axis has been shown to act on Runx2 in bone marrow mesenchymal stem cells, and they are presented separately here for that reason.

Additionally, MALAT1 regulates osteogenic differentiation in periodontal ligament stem cells by binding miR-93-5p, which relieves the repression of SMAD5, an important mediator of bone morphogenetic protein signalling, and therefore raises rather than lowers SMAD5 activity. Knockdown of MALAT1 leads to decreased osteogenic markers and mineralization while miR-93-5p inhibition reverses the effects, suggesting that MALAT1 enhances osteogenesis through the miR-93-5p/SMAD5 axis (22).

The ceRNA effect by MALAT1 is not limited to these miRNAs, as MALAT1 also regulates miR-129-5p in BMSCs, which regulates downstream genes and regulates osteogenic differentiation. In that study Malat1 was higher and miR-129-5p lower in bone marrow stem cells from aged mice than from young mice. Inhibiting Malat1 reduced osteogenic differentiation, and the reduction was reversed by lowering miR-129-5p, which places this axis in the same direction as the others in this section: Malat1 supports osteogenic differentiation by sequestering miR-129-5p (23).

Importantly, the MALAT1 mediated ceRNA network is an alternative pathway for osteogenesis to the well-established Wnt/β-Catenin pathway. This will add to knowledge of MALAT1 involvement in bone homeostasis and regulation of several miRNA interactions for fine-tuning the expression of important transcription factors involved in osteogenesis. The interactions differ in how well they are supported. MALAT1 binding to miR-30, miR-96, miR-93-5p and miR-129-5p, with derepression of Runx2, Osterix, SMAD5 and the osteogenic programme of aged stem cells respectively, has been tested by reporter or immunoprecipitation assay together with a functional readout in skeletal or dental cells. The miR-214 and miR-204 interactions are supported in other settings, as set out above. These should not be presented as a single uniform mechanism.

MALAT1, overall, is a miRNA sponge that can sequester a number of miRNAs that are known to regulate important transcription factors involved in osteogenesis, such as Runx2, Osterix and Dlx5. In addition, this ceRNA mechanism can be exploited to promote the osteogenic differentiation of different types of MSCs, and it can also act as a regulatory axis, in the absence of β-Catenin, to expand its potential role in bone biology and thus offer new targets for bone diseases like osteoporosis and fracture healing (17, 18, 22, 23). As illustrated in Figure 2**,** MALAT1 plays a role in the regulation of osteogenic and osteoclastogenic transcription factors by binding to a number of miRNAs, forming a complex ceRNA network. This expands the mechanism of MALAT1 in bone homeostasis to uncover a post-transcriptional regulatory pathway that is independent of but parallel to the β-catenin-OPG/Jagged1 axis.

**Figure 2:**
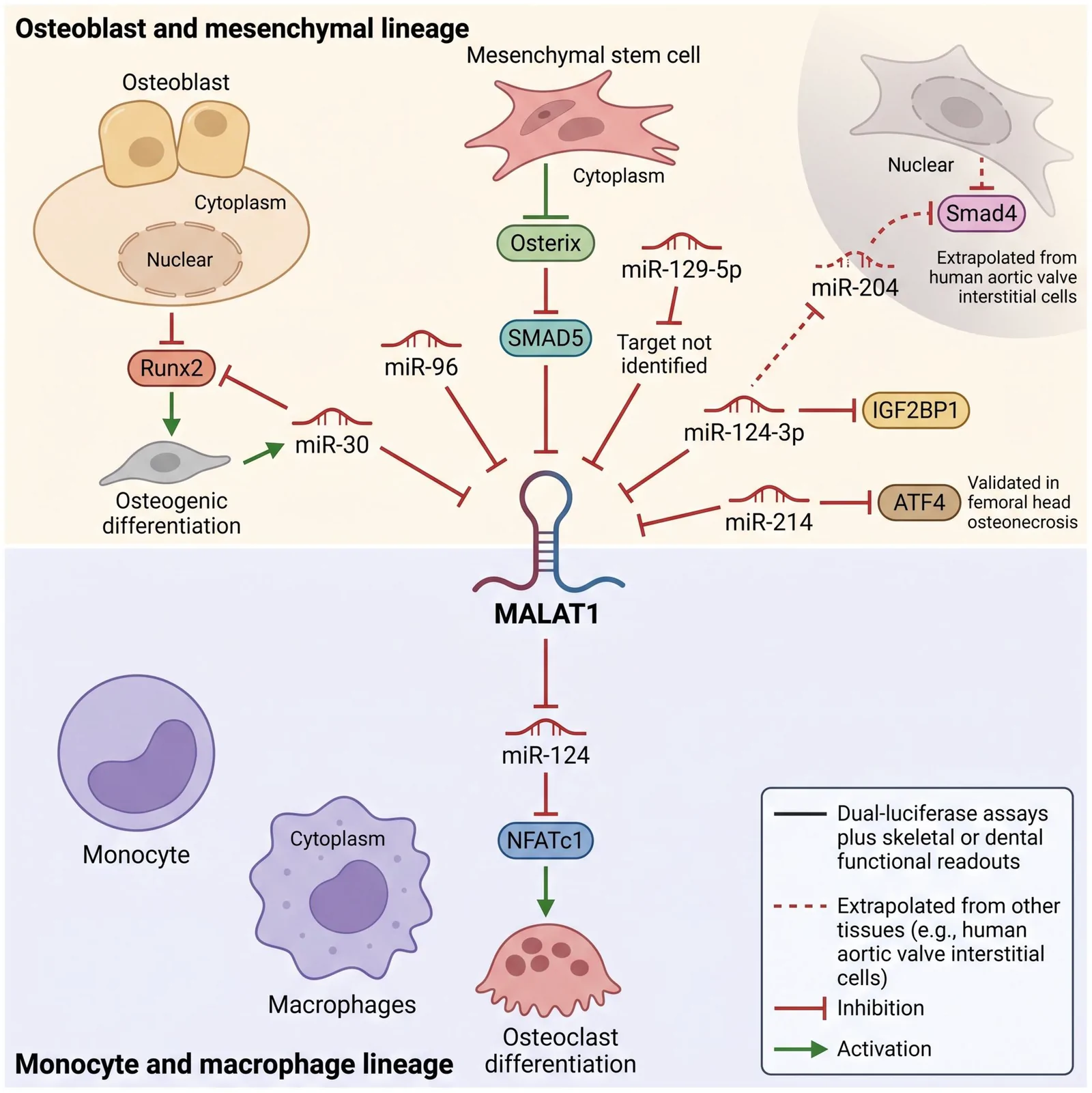
MALAT1 as a competing endogenous RNA (ceRNA) network regulating osteogenic and osteoclastic differentiation. MALAT1 sequesters individual microRNAs and relieves their repression of the corresponding target transcript. In the osteoblast and mesenchymal lineage, sequestration of miR-30 derepresses Runx2, of miR-96 derepresses Osterix, of miR-93-5p derepresses SMAD5, of miR-129-5p targets an unidentified transcript, of miR-124-3p derepresses IGF2BP1 upstream of Wnt/β-catenin, and of miR-214 derepresses ATF4. The miR-204 arm is drawn dashed because the only MALAT1/miR-204 osteogenic study was performed in human aortic valve interstitial cells rather than in skeletal cells, and its target is Smad4. In the monocyte and macrophage lineage, sequestration of miR-124 derepresses NFATc1 and favours osteoclast differentiation. The same transcript appears on both sides of the figure, which is why the net skeletal effect of MALAT1 depends on the cell type in which it is manipulated rather than being fixed in one direction. Solid connectors mark interactions confirmed by dual-luciferase reporter assay together with a functional readout in skeletal or dental cells; dashed connectors mark interactions extrapolated from other tissues; blunt-ended connectors denote repression.

Two general limitations of ceRNA biology apply to all of the interactions described above. Competition for a microRNA is only meaningful when the competing transcript is present at a concentration comparable to that of the microRNA and of its other targets; at the copy numbers typical of most long non-coding RNAs the predicted derepression is small, and quantitative measurements of microRNA and target abundance are needed before a sponge effect can be assumed (30). Independent appraisals have further shown that reported sponge effects are frequently not reproduced once target abundance is measured rather than inferred, and that transfection-based overexpression can produce derepression that does not occur at endogenous transcript levels (31). Most of the MALAT1 axes summarised here rest on overexpression or knockdown combined with a dual-luciferase reporter, a design that establishes that the two sequences can interact but not that the interaction is stoichiometrically relevant in bone cells under physiological conditions. These axes should therefore be read as candidate mechanisms whose quantitative contribution to bone remodeling remains to be established.

### 4.2 MALAT1 regulates osteoclast differentiation via miRNA sponging

Long non-coding RNA MALAT1 is significant involved in osteoclast differentiation, primarily by functioning as a competitive endogenous RNA (ceRNA) to competitively bind certain microRNAs (miRNAs) which are key regulators of osteoclastogenesis. The axis for which direct evidence exists is MALAT1 and miR-124. In bone marrow derived macrophages and in RAW264.7 cells, MALAT1 binds miR-124 and relieves its repression of nuclear factor of activated T-cells cytoplasmic 1 (NFATc1), the transcription factor that drives osteoclast differentiation. The interaction was confirmed by dual-luciferase reporter assay and the functional consequence by reciprocal knockdown and rescue, which makes this the best supported ceRNA interaction of MALAT1 in the osteoclast lineage (15, 32).

*In vivo* this pro-osteoclastogenic effect is opposed by osteoprotegerin, the decoy receptor for RANKL produced by osteoblast lineage cells, so that the net resorptive outcome depends on the RANKL to OPG ratio in the surrounding tissue rather than on the MALAT1 level in the precursor alone. The OPG pathway is a negative feedback system that helps to regulate the process of osteoclast differentiation, which is stimulated by MALAT1, and helps to maintain bone homeostasis.

The ceRNA network of MALAT1 is highly complex and context-dependent, with MALAT1 having the ability to regulate both positively and negatively. The overall effect MALAT1 has on osteoclastogenesis is dependent on the cell environment and microenvironmental cues, because of the complex interaction between miRNA and mRNA. In bone marrow derived macrophages, for instance, MALAT1 binds miR-124 and raises NFATc1, which enhances osteoclastogenesis when the macrophages are exposed to osteoblast-derived exosomes (15, 32). Where MALAT1 appears to reduce osteoclast differentiation, the effect has been observed when MALAT1 is manipulated in osteoblast lineage or mesenchymal cells rather than in the precursor itself, and it is mediated by the RANKL to OPG ratio those cells present. We are not aware of a study in which raising MALAT1 within an osteoclast precursor reduces its differentiation.

The same microRNA family appears on the osteogenic side of the network: MALAT1 sequesters miR-124-3p and thereby raises IGF2BP1, which activates Wnt/β-catenin signalling in bone marrow stem cells (14). The contrast between that result and the macrophage result above is the clearest illustration in this review that the direction of the MALAT1 effect is set by the cell type rather than by the transcript. The role of MALAT1 in influencing both the osteoclast and osteoblast lineage is highlighted in this axis, demonstrating MALAT1's potential to regulate bone homeostasis.

To summarise, the ceRNA contribution of MALAT1 to osteoclast differentiation rests on the miR-124/NFATc1 axis, which is supported by reporter and rescue experiments in macrophage lineage cells. Such interactions are finely orchestrated and there are compensation mechanisms, such as OPG signaling, and they are cell-context dependent, therefore, MALAT1 is important for bone resorption/remodelling (14, 15, 32). Table 1 includes all the molecular mechanisms by which MALAT1 can participate in bone homeostasis, such as β-catenin/OPG/Jagged1 signaling, ceRNA networks, MSC fate determination, immune modulation and exosome-mediated communication. It is, therefore, crucial to stress the complexity of the regulation of MALAT1 function and its key role in orchestrating bone remodeling in these aspects.

**Table 1:** Summary of MALAT1 regulatory mechanisms in bone homeostasis.

Regulatory Mechanism	Molecular Axis/Interaction	Downstream Effectors	Effect on Bone Homeostasis	Key References	Evidence level
β-Catenin-OPG/Jagged1 signaling	MALAT1 binds β-catenin → nuclear translocation ↑	OPG (inhibits RANKL); Jagged1 (activates Notch)	Osteogenesis ↑; Osteoclastogenesis ↓	(13)	Animal *in vivo*: global and osteoblast-conditional Malat1 knockout mice, with supporting cell culture work. No human data.
ceRNA: osteogenic miRNAs	MALAT1 sponges miR-30, miR-96, miR-93-5p	Runx2, Osterix, SMAD5	Osteoblast differentiation ↑	(17, 18, 22)	*In vitro* only, in human and rodent mesenchymal and stem cell culture. Patient-derived cells show the expression difference, but there is no *in vivo* or interventional human evidence.
ceRNA: osteoclastogenic miRNAs	MALAT1 sponges miR-124	NFATc1	Osteoclast differentiation ↑	(15, 32)	*In vitro* in macrophage lineage cells, plus one mouse fracture model. No human data.
MSC fate determination	MALAT1 sponges miR-129-5p, miR-485-5p	Runx2, WNT7B	Osteogenic vs. adipogenic balance	(23, 34)	*In vitro*, using cells from aged mice, plus one mouse hindlimb-unloading model. No human data.
Macrophage polarization	MALAT1 sponges miR-30b; suppresses M2 polarisation	M2 markers (IL-10, Arg1) restrained; M1 markers unchanged	Pro-inflammatory macrophage state; effect on bone repair not measured	(35)	*In vitro*, mouse ligature-induced periodontitis *in vivo*, and observational expression in human gingival tissue. No skeletal readout was measured.
T cell-bone cell crosstalk	MALAT1 modulates NFAT, NF-κB; promotes Th17/Treg balance	RANKL, IL-17, IL-10	Indirect regulation of osteoclastogenesis	(36–38)	*In vitro* and mouse *in vivo*, but in non-skeletal models: T cell memory, viral myocarditis and cardiac allograft. The effect on bone is inferred, not measured.
Exosome-mediated communication	MALAT1 shuttled via exosomes from osteoblasts/MSCs	miR-124/NFATc1; miR-34c/SATB2	Intercellular signaling; osteoclastogenesis ↑/osteogenesis ↑	(15, 16, 32)	*In vitro*, plus rodent osteoporosis and fracture models. Human evidence is observational expression only.

The regulation of bone homeostasis by MALAT1. The major molecular pathways of bone remodeling can be subdivided by “regulatory type”, “molecular axis”, “downstream effectors”, and “biological outcomes” and are represented by MALAT1. The basic signaling pathway leading to the activation of the β catenin is OPG/Jagged1 axis. The ceRNA mechanism in osteogenic and osteoclastogenic differentiation is regulated at multiple levels of post transcriptional regulation with multiple miRNAs playing a role. MALAT1 also plays a role in cell fate of MSC, macrophage polarization, T cell/bone communication and in intercellular communication by exosomes. Taken together, these pathways position MALAT1 as a regulatory component acting at several points in bone homeostasis. The level of evidence supporting each mechanism is given in the final column.

The opposing effects attributed to MALAT1 in this section and in Section 3 require a direct explanation rather than a restatement, and three variables account for most of the discrepancy. The first is the cell source in which MALAT1 is manipulated. Studies reporting that MALAT1 drives osteoclastogenesis act on monocyte-macrophage lineage cells, RAW264.7 cells or bone marrow derived macrophages, in which the transcript is altered within the precursor itself (15, 32). Studies reporting the opposite direction manipulate MALAT1 in osteoblast lineage or mesenchymal cells and read out osteoclast behaviour indirectly, through the RANKL to OPG ratio that those cells present (14). The second variable is the stimulation condition. Pro-osteoclastogenic effects are observed under sustained RANKL or exosome challenge in culture, where the opposing OPG arm is largely absent from the system; when the same axis is examined *in vivo*, OPG produced by osteoblasts buffers it, which is why global Malat1 deletion raises osteoclast number and surface even though Malat1 does not act on osteoclasts cell-autonomously (13). The third variable is disease context, since in multiple myeloma the modified exosomal transcript reaches osteoclast precursors from tumour cells rather than from resident bone cells, and the relevant comparison is with the tumour rather than with normal remodeling (33). Read together these results are compatible rather than contradictory: MALAT1 is pro-osteoclastogenic when acted on cell-autonomously within the precursor, is net anti-resorptive when acted on in the osteoblast lineage, and the phenotype observed *in vivo* reflects the second of these.

## 5 The regulatory role of MALAT1 in osteogenic differentiation of bone marrow mesenchymal stem cells

### 5.1 MALAT1 regulates mesenchymal stem cell self-renewal and osteogenic fate determination

MALAT1 regulates important pathways in the bone marrow mesenchymal stem cells (MSCs) including β-Catenin and Notch pathways that play a role in the regulation of stemness and osteogenic differentiation of MSCs. The expression of pluripotency markers such as Oct4 and Sox2 is critical to MSC self-renewal and multipotency and these pathways are essential. Malat1 has been consistently upregulated during osteogenic induction of MSCs and Malat1 upregulation has been shown to be associated with upregulation of osteogenic transcription factors such as Runx2 and Osterix (Osx) as well as osteogenic markers such as osteocalcin (OCN), osteopontin (OPN) and activity of alkaline phosphatase (ALP) (17, 18, 39). Just as a competing endogenous RNA (ceRNA) can bind to microRNAs (miRNAs) like miR-30, miR-96, miR-124 and miR-214-3p, freeing up the target genes, similar action is observed here. For instance, MALAT1 binds to the miR-30 and the resultant binding regulates the expression of Runx2, which is a master regulator in the osteogenic lineage differentiation of adipose-derived MSCs, thus resulting in an increase in the osteogenic commitment of MSCs (17). Likewise, MALAT1 regulates miR-96/Osx axis in human bone marrow-derived MSCs (hBMSCs) which promote the osteogenic differentiation by upregulating Osx, which is a key transcription factor downstream of Runx2 (18, 40). Moreover, MALAT1 can modulate the miR-124/IGF2BP1/Wnt/β-catenin pathway and promote osteogenesis and inhibit osteoclastogenesis, suggesting its involvement in bone homeostasis and it could be a therapeutic target for osteoporosis (14). In addition, Notch signaling pathway, controlled by MALAT1, which induces the expression of Notch pathway targets and favors MSC proliferation and differentiation to their osteogenic subpopulation (39) was also implicated.

Knockdown MALAT1 led to the redirection of MSC differentiation from osteogenesis to adipogenesis as reflected by the increased expression of the adipogenic transcription factors PPARγ and C/EBPα, and the decreased expression of osteogenic markers, suggesting that MALAT1 is crucial for maintaining the balance between the osteoblast and adipocyte lineages (41, 42). The balance is crucial for bone integrity, with an upregulation of differentiation of adipocytes in the bone marrow niche associated with bone mass decrease and osteoporosis. Mechanical cues could play a role in regulating MALAT1 expression since MALAT1 regulates adipocyte differentiation in a biophysical manner. MALAT1 expression has been shown to be under the regulation of mechanical stimuli and MALAT1 is able to regulate the differentiation of adipocytes, thus connecting mechanical cues with MALAT1-mediated lineage specification (42).

Epigenetic regulation is another mechanism involved in MSC fate regulation by MALAT1. MALAT1 is a long non-coding RNA, not a protein, and it has been reported to associate with EZH2, the catalytic subunit of Polycomb Repressive Complex 2, which deposits the trimethylation mark on lysine 27 of histone H3 (43). That mark is repressive: it compacts chromatin and silences the associated genes rather than opening them. The consequence for osteoblast differentiation therefore depends on which loci are targeted, and whether MALAT1 directs PRC2 to osteogenic loci in bone cells has not been established. Additionally, MALAT1 can work with a number of RNA-binding proteins, including KHSRP, to regulate mRNA stability of key osteogenic regulators, such as RUNX1, through the regulation of ubiquitination by NEDD4, which ultimately promotes osteogenesis (44).

MALAT1 is also part of the intercellular communication in the bone microenvironment. The PROs derived from the MSCs with the MALAT1 have been proved to improve the osteoblast activity through the regulation of miR-34c/SATB2 axis, which leads to bone formation in osteoporotic models (16). The results of the exosomal transfer indicate that MALAT1 can play a role in the intracellular regulation as well as in paracrine regulation of bone remodeling.

Pathological conditions like osteoporosis and osteonecrosis usually result in downregulation of MALAT1 expression that is related to the reduced capacity of osteogenic differentiation and enhanced adipogenesis of MSCs (41, 45). MALAT1 has been reported to be related to bone loss; and the restoration or modulation of downstream miRNA targets have been reported to restore osteogenic potential *in vitro*, indicating MALAT1 as a potential therapeutic target. MALAT1, on the other hand, inhibited osteogenic differentiation of PDLSCs via miR-155-5p/ETS1 axis, suggesting that inflammatory signaling and bone regeneration is correlated in inflammatory diseases such as periodontitis (46).

In summary, these results indicate that MALAT1 can perform complex functions in the regulation of MSC self-renewal and osteogenic differentiation through miRNA sponging, epigenetic regulation, activation of signaling pathways (β-Catenin, Notch, Wnt) and intercellular communication via exosomes. The regulation of the balance between osteogenesis and adipogenesis is critical to bone homeostasis, and is a mechanism in bone-related diseases. The identification of Malat1 regulatory networks and interactions with other lncRNAs and transcription factors will help to better understand the role of Malat1 in skeletal biology, and identify novel therapeutics for bone regeneration and osteoporosis therapy through Malat1 (17, 39, 43).

### 5.2 MALAT1 and its synergistic interaction with microenvironmental factors

The long non-coding RNA (lncRNA) or metastasis-associated lung adenocarcinoma transcript 1 (Malat1) is recognized as a key player in linking various signals in the microenvironment with the regulation of osteogenic differentiation of mesenchymal stem cells (MSCs) and, hence, bone homeostasis. Critical microenvironmental cues that dynamically regulate MALAT1 expression, which in turn orchestrates downstream osteogenic pathways, are mechanical stress, hypoxia and inflammatory cytokines. Mecano-transduction pathways, such as YAP/TAZ signaling axis, have been demonstrated to induce MALAT1 expression in response to mechanical stimuli, like the cyclic stretching and the fluid shear stress. For example, MALAT1 was found to be up-regulated under the physiologically relevant shear stress in MSCs and the change in MSC morphology and osteogenic differentiation indicate MALAT1 to be a mechanosensitive lncRNA contributing to the enhanced anabolic response to mechanical loading in bone formation (42). This mechanosensitive regulation is important as mechanical loading is known to promote osteogenesis, and the upregulation of MALAT1, in response to mechanical loading, promotes the transcription activation of osteogenic genes, which increases MSC commitment to the osteoblastic lineage.

In contrast, inflammatory cytokines [e.g., tumor necrosis factor alpha (TNF-α)] that are abundant in pathological bone microenvironments in which chronic inflammatory conditions are present have inhibitory effects on MALAT1 expression via activation of NF-κB signaling pathways. When NF-κB is stimulated by TNF-α, MALAT1 is silenced and this reduces the osteogenic potential of MSCs. This is shown in inflammatory bone diseases and steroid-induced osteonecrosis, where the expression of important osteogenic markers, including RUNX2, ALP, and osteocalcin (OCN) are diminished, as is expression of MALAT1 in these diseases (28). The intricate mechanical and inflammatory regulation highlights MALAT1 as a molecular integrator that transduces the extracellular microenvironmental cues into distinct gene expression programmes that control Osteogenesis.

Hypoxia is a further feature of the marrow niche that has been associated with changes in MALAT1 expression, but the mechanism remains incompletely understood and should not be presented as established. A direct interaction between hypoxia-inducible factors and regulatory elements of the MALAT1 locus has been proposed rather than demonstrated in bone cells, and the available skeletal evidence is limited to the observation that hypoxia induces MALAT1 and that the miR-22-3p/CEBPD axis operates downstream during fracture healing (47). Whether hypoxia shifts the balance between osteogenic and adipogenic commitment through MALAT1, or through the several HIF-dependent pathways that act on that balance independently of it, has not been resolved. The role of hypoxia, MALAT1 and MSC fate decisions is very interesting, particularly for the compromise of bone mass and integrity by the excessive adipocyte differentiation in the bone marrow microenvironment (42).

All these findings suggest that MALAT1 is an important molecule that acts as a cross linker of mechanical, inflammatory and hypoxic signals in the bone microenvironment to allosterically regulate MSC osteogenic differentiation. MALAT1 serves as a molecular hub to connect extracellular stimuli to intracellular signalling networks and transcription networks, including those involving β-Catenin-OPG/Jagged1, which are known to be important for bone homeostasis. This integrative role of MALAT1 suggests that MALAT1 could be a therapeutic target for bone disorders in which there is a disruption in the bone microenvironmental signaling, including osteoporosis, steroid-induced osteonecrosis, and inflammatory bone diseases, and also helps to broaden the understanding of bone biology. It is important to understand the detailed molecular mechanisms by which the MALAT1 is regulated by microenvironmental factors and its downstream effectors for developing interventions based on MALAT1 to restore or enhance bone formation under pathological conditions.

## 6 The function of MALAT1 in osteoclast differentiation and bone resorption

### 6.1 MALAT1 regulation of osteoclast precursor cell proliferation and differentiation

A highly conserved long non-coding RNA (lncRNA), metastasis-associated lung adenocarcinoma transcript 1 (MALAT1) is a key factor in bone remodeling, especially in the proliferation and differentiation of osteoclast precursor cells. It is demonstrated that RAW264.7 cells and bone marrow-derived macrophages (BMMs) are able to differentiate into mature osteoclasts when stimulated by receptor activator of nuclear factor-kappa-B (RANK) ligand (RANKL). MALAT1 expression was significantly induced after stimulation with RANKL, indicating its role in the process of osteoclastogenesis. MALAT1 is a key player in mechanistically promoting osteoclast differentiation by inducing the activation of key signaling pathways such as NF-κB and mitogen-activated protein kinase (MAPK) cascades involved in the regulation of proliferation and maturation of osteoclast precursor cells. For instance, endothelial progenitor cell (EPC)-derived exosomes that are highly enriched with MALAT1 promote the migration of BMMs and osteoclastogenesis by directly binding MALAT1 and down-regulating the expression of miR-124, which is a microRNA that inhibits osteoclastogenesis. The MALAT1-miR-124 interaction is thought to lead to expression of the integrin ITGB1, which encourages the recruitment and differentiation of osteoclast precursors (32). Similarly, under co-cultivation, when RAW264.7 cells are co-cultivated with the breast cancer cells, MCF-7 cells, the expression of MALAT1 is upregulated in RAW264.7 cells, and results in spontaneous osteoclastogenesis via the RANKL/MALAT1/miR-124 axis. In addition, the level of miR-124 was found to be increased by denosumab, a RANKL inhibitor, and denosumab decreased the expression of MALAT1 demonstrating the crucial role of MALAT1 in the inhibition of osteoclastogenesis induced by RANKL (48).

Further, through functional experiments, it is confirmed that MALAT1 positively promotes osteoclast differentiation. Knocking down MALAT1 expression in osteoclast precursor cells leads to a significant decrease in osteoclastogenesis, as measured by the number of tartrate-resistant acid phosphatase (TRAP)-positive multinuclear cells, disruption of actin ring assembly and loss of bone resorption pit area. The phenotypic modifications illustrate the critical function of MALAT1 for both the organization of the cytoskeleton, as well as the resorptive process of mature osteoclasts (15). Secondly, the osteoblast-exosomes carry MALAT1 that has been reported to be a sponge for miR-124, which in turn regulates NFATc1, a master transcription factor for osteoclast differentiation. Both MALAT1 knockdown and miR-124 overexpression have been shown to lead to downregulation of NFATc1 expression and to inhibit osteoclast formation, while miR-124 inhibition has been shown to restore NFATc1 expression and osteoclast formation, indicating that the MALAT1/miR-124/NFATc1 signaling axis is crucial in osteoclast precursor differentiation (15).

MALAT1 appears to be pro-osteoclastogenic *in vitro*, its action *in vivo* is regulated by the antagonist of RANKL, called osteoprotegerin (OPG). The OPG signaling is, however, balanced by the MALAT1 signal, which stimulates osteoclast differentiation and bone resorption, thus creating a negative feedback regulation of bone resorption in the physiological range. This regulatory circuit helps to keep bone homeostasis even when there is too much activity of the osteoclasts and therefore pathological bone loss. Of particular interest, Malat1 knockout mice develop osteoporosis that is not the result of a direct effect on osteoclasts. The phenotype arises from impaired osteoblast function and from disrupted communication between osteoblasts, osteoclasts and chondrocytes through the β-catenin-OPG/Jagged1 pathway, which the authors of that study demonstrated using an osteoblast-specific conditional knockout (13). This highlights MALAT1 as a crucial player in bone remodeling networks and the ability to regulate signals to influence osteoclast precursor proliferation and differentiation in the bone microenvironment.

Moreover, NSUN2-dependent methylation of MALAT1 raises its stability in multiple myeloma cells, and the modified transcript is exported in exosomes to osteoclast precursors, where it induces RANKL expression and activates AKT and MAPK signalling (33). This route is specific to the tumour setting: the transcript reaches the precursor from myeloma cells rather than from resident bone cells, so it should not be read as a description of normal remodeling.

Finally, MALAT1 is shown to be positively regulated in osteoclast precursor cells in response to RANKL, to activate NF-κB and MAPK pathways, and to regulate key transcription factors (NFATc1) by miRNA sponging mechanisms, in conclusion providing a positive regulatory role in osteoclastogenesis. *In vivo*, its pro-osteoclastogenic properties are physiologically well regulated by negative feedback by OPGs to maintain bone resorptive activity within a physiological range. MALAT1 is also involved in complex intercellular interaction through exosomes which affect the recruitment and differentiation of osteoclasts both in normal and pathological conditions. These findings indicate that MALAT1 is an important regulatory component of osteoclast precursor proliferation and differentiation, with implications for bone homeostasis and for diseases associated with abnormal bone resorption.

### 6.2 MALAT1 regulates osteoclast function through cytoskeletal modulation

In addition to its transcription factor activity, the long non-coding RNA MALAT1 has been shown to be an important regulator of the function of osteoclasts, including the regulation of bone resorptive cytoskeletal dynamics. Reorganisation of the actin cytoskeleton underlies the sealing zone and the F-actin ring, the structures an osteoclast requires in order to degrade bone matrix, and knockdown of MALAT1 disrupts them (15). MALAT1 has been linked to signalling pathways and genes that govern cytoskeletal organisation, including the RhoA/ROCK pathway and the actin polymerisation regulators CDC42 and WASL. The strength of this link differs by system and the distinction matters here: the RhoA/ROCK and CDC42/WASL connections to MALAT1 were established in non-skeletal cells, and we are not aware of a study that manipulates MALAT1 in osteoclasts and measures these effectors directly. What has been shown in osteoclasts is that MALAT1 knockdown disrupts F-actin ring formation and reduces resorptive capacity, so the cytoskeletal phenotype is bone-specific evidence while the proposed molecular route to it is an extrapolation. The role of RhoA/ROCK signaling axis in regulating actin cytoskeleton rearrangement, cell polarity and cell motility is well established and all three are essential for osteoclast polarization and bone resorptive activity. Actin filaments are part of the osteoclast and are regulated by the spatial organization of the actin filaments regulated by MALAT1 and this pathway.

In osteoclasts, MALAT1 knockdown disrupted actin ring assembly and reduced the area of resorption pits alongside a fall in the number of TRAP-positive multinucleated cells (15). That is the bone-specific evidence, and it establishes a cytoskeletal phenotype rather than a molecular route to it. A role for MALAT1 in regulating CDC42 and WASL has been proposed by analogy with non-skeletal systems, but we could not identify a study that measures either effector in osteoclasts after manipulating MALAT1, so it is not asserted here.

This expanded knowledge of MALAT1's osteoclast function adds to what was previously known about its well established role in transcription regulation, thereby supporting the role of MALAT1 in direct cytoskeletal remodeling of osteoclasts. Whether MALAT1 acts on the RhoA/ROCK pathway and the actin polymerisation machinery in osteoclasts specifically has not been tested, and the connection to osteoclast polarisation, migration and resorption is therefore proposed rather than demonstrated. This additional layer of control adds to the regulatory role of MALAT1 in osteoclast activity, which may have therapeutic implications in bone diseases where there is a high osteoclast activity, like osteoporosis. Therefore, modulation of MALAT1 expression or function might be a new therapeutic approach to restore bone homeostasis by correcting the abnormal structure of the cytoskeleton in osteoclasts to allow for the attenuation of pathological bone loss.

## 7 The role of MALAT1 in immune regulation of the bone microenvironment

The material in this section is included because the immune and skeletal systems share regulators, cytokines and precursor populations rather than because MALAT1 has general immune functions. Osteoclasts differentiate from the monocyte-macrophage lineage, RANKL is produced by activated T cells as well as by osteoblasts, and the cytokine output of T cell subsets acts directly on osteoclast differentiation, which is the basis of osteoimmunology as a field (49). The subsections below are therefore restricted to those MALAT1-dependent immune mechanisms for which a skeletal readout has been reported or for which the effector cytokine has an established action on bone; general immune phenotypes are cited only where they identify the molecular step involved.

### 7.1 MALAT1 regulation of T cell and bone cell interactions

The long non-coding RNA (lncRNA) MALAT1 (Metastasis Associated Lung Adenocarcinoma Transcript 1) plays an important role in modulating the communication between T cells and bone cells, thereby indirectly affecting osteoclastogenesis and bone resorption in the bone immune microenvironment. This regulatory role is mainly exerted by MALAT1 on the secretion of crucial cytokines [receptor activator on nuclear factor kappa-Β ligand (RANKL) and interferon-gamma (IFN-γ)] by T cells. RANKL is crucial for the differentiation and activation of osteoclasts, while IFN-γ may have inhibitory effects on osteoclastogenesis and has more complex role in bone remodeling. Activated T cells produce RANKL and thereby contribute to osteoclast differentiation, which is a well established element of osteoimmunology (49). MALAT1 is induced during T cell activation and shapes the cytokine output of those cells (36), so a route from MALAT1 through NFAT to RANKL is plausible. We were unable to identify a study that measures RANKL or a bone outcome after manipulating MALAT1 in T cells, and the step is therefore presented as proposed rather than demonstrated. In addition, MALAT1 also regulates the differentiation of regulatory T cells (Tregs) that play a role in maintaining immune homeostasis and bone stability. MALAT1 is involved in the regulation of immune response to bone remodeling by modulating the secretion of anti-inflammatory cytokines such as interleukin-10 (IL-10) and transforming growth factor-β (TGF-β) to ensure the balance between two cell types (osteoclasts and osteoblasts) and bone integrity.

The MALAT1 is involved in some microRNAs (miRNAs) that target the mRNA of activated cells mediated by T lymphocytes, and also acts as a molecular sponge for some microRNAs, that target the mRNAs associated with cytokine production. For example, MALAT1 binds to miR-15/16 in cytotoxic T cells and upregulates the expression of CD28 and interleukin-2 (IL-2), which plays an important role in the activation and proliferation of T cells. The interaction between MALAT1 and miR-15/16 is crucial for maintaining the activation and memory of cytotoxic T cells, so disruption of this interaction might also affect bone resorption by impairing activation and memory formation of T cells, and indirectly via cytokines (36). In addition, MALAT1 has the ability to regulate NF-kB signaling pathways in immune cells, such as dendritic cells and T cells, as well as the transcription of co-inhibitory molecules such as CD80, and the secretion of inflammatory cytokines. The same holds true for the bone microenvironment in T cell mediated immune response (50).

It has previously been suggested that MALAT1 is involved in bone pathologies in inflammatory diseases such as autoimmune disorders and acute viral myocarditis, and has been implicated in the differentiation and activity of CD4+ T cells of the pro-osteoclastogenic T helper 17 (Th17) subset. Oral inhibition of MALAT1 is capable of blocking the polarization of Th17 cells and inflammation, thus indicating that MALAT1 could play a role in the inflammatory environment which promotes osteoclastogenesis and bone loss (37). Moreover, MALAT1 can also exert a regulatory effect on the Tregs via the miR-155/DC-SIGN/IL-10 pathway in DCs, which can be relevant for bone homeostasis by regulating T cell subsets and cytokine production (38).

Further, the immunomodulatory role of MALAT1 has been confirmed in the tumor immune microenvironment, as it is expressed in T cells and tumor-associated macrophages (TAMs) and has an impact on immune escape and the activity of cytotoxic T cells (CTLs). Inhibition of MALAT1 increases the viability of CD8+ T cells and the production of inflammatory cytokines, which suggests that MALAT1 could play a role in the bone metastasis of cancer and bone remodeling (51). In addition, MALAT1 can bind to microRNAs (miR-195 and miR-124-3p) to regulate the expression of immune checkpoints and T-cell cytotoxicity and immune escape pathways (52) that could influence the integrity of the bone in malignancies.

All taken together, these results suggest that MALAT1 plays an important role in the function and differentiation of T cells within the bone immune niche. MALAT1 has been shown to indirectly regulate osteoclastogenesis through the regulation of the secretion of osteoclastogenic and immunoregulatory cytokines such as NFAT and NF-κB, and by influencing the balance of T cells subsets. This suggests that MALAT1 may be an immunoinflammatory bone disease and abnormal bone remodeling of immune cells drug target. The molecular mechanisms must still be deciphered and therapeutic strategies targeting MALAT1 to restore the bone homeostasis by regulating the communication between T cells and bone cells should be investigated.

### 7.2 MALAT1 in macrophage polarization and bone repair

Long noncoding RNA MALAT1 (metastasis-associated lung adenocarcinoma transcript 1) has been revealed to be a key mediator that regulates polarization of macrophages and to play a key role in the inflammatory milieu and therefore the subsequent bone repair processes. Macrophages are plastic cells whose activation states are conventionally summarised as pro-inflammatory M1 and anti-inflammatory M2. That binary is a simplification retained here only for comparability with the cited studies. Macrophage activation is better described as a spectrum of overlapping states determined by the specific stimulus, and published guidance recommends that activation be reported by the activating condition and the markers measured rather than by the M1 or M2 label alone (53). The studies discussed below each use a restricted marker set, and their labels should be read accordingly. The regulatory actions of MALAT1 include the ability to regulate the relationship between these macrophage phenotypes and thus to control the inflammatory response and promote bone regeneration. MALAT1 expression is dynamically regulated during the differentiation and activation of macrophages and several studies have demonstrated it. In the study that defined this axis, MALAT1 knockdown increased M2-associated markers without changing M1-associated markers, which the authors interpreted as MALAT1 favouring the M1 state by suppressing M2 differentiation; in human periodontal tissue and in ligature-induced periodontitis in mice, higher MALAT1 accompanied higher M1 markers and lower miR-30b, supporting a pro-inflammatory role *in vivo* (35). This antagonism is observed in the interaction with micro RNAs such as miR-30b, which is involved in polarization to the M2 phenotype, and MALAT1 which acts as a competing endogenous RNA (ceRNA), which binds to miR-30b and avoids polarization towards M2 phenotype, maintaining inflammatory functions, as observed (35). The balance between M1 and M2 macrophages in bone repair is important because M1 macrophages trigger inflammation, which is needed to clear debris and M2 macrophages produce anti-inflammatory cytokines (IL-4 and IL-10), which promote osteogenic differentiation and tissue remodeling.

A contrary direction has been reported in tumour and tissue-injury settings, and the discrepancy is real rather than apparent. Extracellular vesicles from triple-negative breast cancer cells deliver MALAT1 to macrophages and promote M2 polarisation through POSTN and the Hippo/YAP axis (54), whereas in the myeloid-intrinsic setting described above MALAT1 restrains M2 differentiation (35). The two systems differ in the source of the transcript, vesicle-delivered vs. endogenous, and in the surrounding cytokine milieu. Whether MALAT1 promotes or restrains M2 polarisation in a healing bone defect specifically has not been tested directly, and the reader should not assume either direction for that setting. The MALAT1 shifted macrophages contribute to building a bone immune microenvironment conducive to bone regeneration. The mechanism is based on the regulation of important signaling pathways and gene expression networks in macrophages by MALAT1. For instance, MALAT1 was identified as a cargo transported by EVs to macrophages where it promotes their M2 polarization through upregulation of POSTN, which leads to activation of Hippo/YAP pathways that are responsible for tissue repair (54). Moreover, MALAT1 is able to control downstream targets including IFIH1 that associates with RNA-binding proteins such as PTBP1 and contribute to the polarization states and inflammatory responses of macrophages (55).

The function of MALAT1 in regulating macrophage polarization is extended to inflammatory diseases and tissue injury conditions, in which it regulates NF-κB signaling pathway and cytokine production. For example, knockdown of MALAT1 inhibits the activation of the nuclear factor-kappaB (NF-κB) and the production of pro-inflammatory cytokines, which reduces inflammation and M1 polarization. This anti-inflammatory activity is crucial in bone repair: An overactive or uncontrolled inflammation and tissue damage can be caused by M1 macrophages, but this may be detrimental to the healing process (6, 56). The corollary is that lowering MALAT1 shifts the balance away from the inflammatory state, which is consistent with the myeloid-intrinsic result described above. It is not evidence that MALAT1 itself drives M2 polarisation, and this section should not be read as making that claim.

In addition to the cytokine modulation, MALAT1 is also a regulator of osteoclastogenesis which controls bone remodeling by macrophages. Osteoclasts arise from the monocyte-macrophage lineage, and in that lineage MALAT1 acts in the direction that favours resorption. In bone marrow derived macrophages, MALAT1 sequesters miR-124 and thereby relieves repression of NFATc1, which promotes rather than restrains osteoclast differentiation (15). Taken with Section 7.2, the picture in the myeloid compartment is consistent: MALAT1 restrains M2 differentiation and promotes osteoclastogenesis, so it acts on both arms in the direction that favours resorption.

Overall, these findings indicate that MALAT1 influences macrophage polarization and, through the inflammatory response, bone regeneration. MALAT1 regulates the bone immune microenvironment by fine-tuning the M1/M2 balance, inducing secretion of anti-inflammatory cytokines, osteogenic differentiation and improved bone healing. The regulatory network makes MALAT1 an interesting therapeutic target since it could help optimize bone repair and be used in the treatment of bone diseases in which inflammation and regeneration is dysregulated. The intricate function of MALAT1 in the regulation of immune cell activation, polarization and modulation of T cell cytokine production and the intercellular interaction through exosomes in the bone microenvironment is summarised in Figure 3. In this context, MALAT1 is highlighted as a central point of integration between inflammation and immunity and bone remodeling.

**Figure 3:**
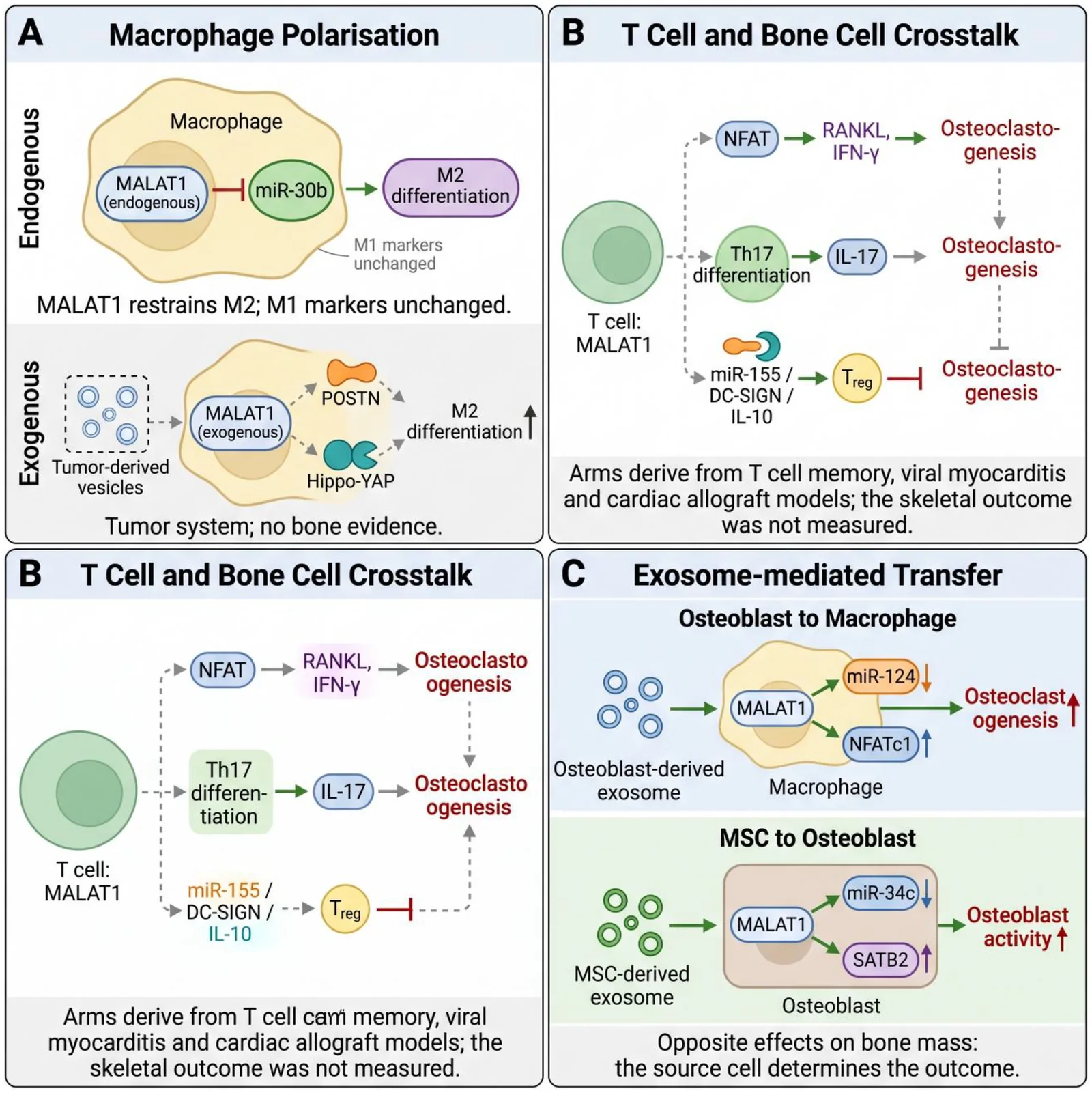
MALAT1 in the bone immune microenvironment: macrophage polarization and T cell-bone cell crosstalk. Three mechanisms are shown in separate panels. **(A)** Macrophage polarisation. Endogenous MALAT1 in macrophages sequesters miR-30b, which is a promoter of the M2 programme; the reported consequence is that M2 differentiation is restrained while M1-associated markers are unchanged, so the endogenous transcript leans the population towards the inflammatory end of the spectrum. The lower row of panel A shows the opposite result obtained with MALAT1 delivered by tumour-derived vesicles, which acts through POSTN and the Hippo/YAP axis; it is drawn dashed because it is a tumour system and is not evidence about bone. **(B)** T cell and bone cell crosstalk. MALAT1 in T cells acts through NFAT to raise RANKL and interferon-gamma, and enhances Th17 differentiation and interleukin-17 output, while the miR-155/DC-SIGN/interleukin-10 axis favours regulatory T cells, which act in the opposite direction. All three arms enter the skeletal outcome as dashed connectors because they were established in non-skeletal models and the bone readout was not measured. (Lower) Exosome mediated communication: Osteoblast-derived exosomal MALAT1 enhances osteoclastogenesis via miR 124/NFATc1 axis in macrophages and MSC derived exosomal MALAT1 enhances osteoblast activity. Through these mechanisms MALAT1 links immune regulatory pathways to bone remodeling.

## 8 Pathological significance of MALAT1 in bone-related diseases

### 8.1 MALAT1 dysregulation and functional impairment in osteoporosis

Osteoporosis is an imbalance between the processes of bone formation and bone destruction resulting in a decrease in bone mass and an increase in fracture risk. In recent years, the long non-coding RNA (lncRNA) MALAT1 has been suggested to play a role as a modulator of bone homeostasis, and may be involved in the pathogenesis of osteoporosis. In human studies, MALAT1 has been found at lower levels in peripheral blood from elderly patients with osteoporosis than in healthy volunteers (14). Correlations between MALAT1 and the circulating bone turnover markers CTX-1 and P1NP have not been reported, and the direction of any such relationship in patients is therefore unknown. MALAT1 regulates the different signalling pathways, molecular axes and modulates osteogenic differentiation in a mechanistic fashion. To recapitulate the features of postmenopausal osteoporosis, MALAT1 overexpression results in an increase in both trabecular number and thickness, thus improving the microarchitecture of the bone, and restoring osteoporotic phenotypes in the case of ovariectomized (OVX) mice (14). This effect is achieved via MALAT1's ability to modulate the differentiation of osteoblasts, and to inhibit osteoclastogenesis, ultimately re-establishing the equilibrium between bone formation and bone resorption. Furthermore, MALAT1 can function as a competing endogenous RNA (ceRNA) to bind the miRNAs, including miR-217, miR-124-3p and miR-485-5p, and sequester the miRNA binding sites of osteogenic regulators, such as AKT3, IGF2BP1 and WNT7B. These interactions can have an impact on the activation of downstream signalling pathways including the Wnt/β-catenin pathway, which is important for both osteoblast function and survival (14, 34, 45). MALAT1 silencing has been shown to decrease the expression of osteogenic markers, including collagen type I, bone morphology protein 4 (BMP4) and alkaline-phosphatase (ALP), and MALAT1 overexpression has the opposite effect, even under inhibitory conditions such as simulated microgravity, *in vitro* using bone marrow-derived mesenchymal stem cells (BMSCs) and osteoblastic cell lines (34, 45). Interestingly, clinical cohort studies have demonstrated the association of serum MALAT1 with osteoporosis diagnosis and monitoring. In addition, MALAT1 was significantly lower in serum from osteoporotic patients compared to healthy controls, and MALAT1 was also correlated with clinical parameters of bone turnover and response to treatment, suggesting that MALAT1 could be a valuable molecule for the non-invasive assessment of the disease (14). Taken together, these data highlight the key role of MALAT1 in bone metabolism as a suppressor of osteoporosis by impairing osteoblast-osteoclast coupling via the β-Catenin-OPG/Jagged1 pathway and other pathways. Restoration of MALAT1 expression or activity is therefore a potential therapeutic strategy for osteoporosis that requires validation. It has so far been tested only in rodent models and in cultured human cells, and no clinical study has assessed it.

### 8.2 Role of MALAT1 in osteoarthritis pathology

In osteoarthritis (OA), a degenerative joint disease involving cartilage degradation, synovial inflammation and subchondral bone remodeling, the long non-coding RNA MALAT1 is found to be important in regulating various pathological processes. Dysregulated MALAT1 expression has been observed in clinical and experimental studies in cartilage and synovial tissues of OA and has several variable and context-dependent functions. The MALAT1 expression has been reported to be increased under inflammatory stimuli, such as interleukin-1β (IL-1β) and lipopolysaccharide (LPS), which mimics the inflammatory microenvironment in OA joints, in articular chondrocytes obtained from animal models and OA patients. Under those conditions MALAT1 acts on the matrix-degrading enzymes MMP-13 and ADAMTS-5, which are the effectors through which cartilage extracellular matrix is turned over (57, 58).

### 8.3 Role of MALAT1 in osteosarcoma

In osteosarcoma, the most common primary malignant bone tumour of children and young adults, MALAT1 is elevated in tumour tissue and in osteosarcoma cell lines, and a high level predicts poorer overall survival, which is the basis for proposing it as a prognostic marker (59). That association is observational, and no interventional study has tested MALAT1 as a therapeutic target in this tumour. On the molecular level, MALAT1 is involved in the molecular regulation of osteosarcoma cells proliferation and invasion through the regulation of important oncogenic pathways like Akt/mTOR and β-Catenin pathways. The Akt/mTOR pathway has been well studied for its role in cell growth, survival and metabolism, whereas the β-Catenin pathway has been shown to regulate the transcription of genes controlling cell proliferation and differentiation. Overexpression of MALAT1 enhances these pathways and fuels the development of aggressive tumour behavior and metastasis.

One mechanism that has been shown for the MALAT1 to promote the oncogenic function in OS is by its function as a competing endogenous RNA (ceRNA). In osteosarcoma cells, MALAT1 acts as a sponge for miR-34a and miR-449, relieving repression of c-Met and SOX4 and promoting proliferation and metastasis (59). EMT gives epithelial tumor cells their mesenchymal properties and leads to their increased motility and invasiveness necessary for metastasis. This ceRNA regulatory axis reveals the intricate post-transcriptional regulatory network in which MALAT1 plays a role in the regulation of the gene networks involved in osteosarcoma metastasis.

Osteoclast hyperactivity drives the osteolytic destruction seen in bone tumours, and a MALAT1-dependent contribution to it has been demonstrated in multiple myeloma, where tumour-derived exosomal MALAT1 induces RANKL in osteoclast precursors (33). We could not identify an equivalent demonstration in osteosarcoma, so whether MALAT1 acts on osteoclasts in that tumour specifically remains untested. The osteoclast-mediated bone degradation is a part of tumour progression to remodel bone niche for tumour growth/invasion.

In summary, these data indicate that MALAT1 is a multifaceted molecule that can modulate osteosarcoma pathogenesis by controlling a variety of cellular processes such as proliferation, invasion, EMT, activation and remodeling of osteoclasts and bone microenvironment. In addition to its role in regulating bone tumor intrinsic pathways (Akt/mTOR and β-Catenin signaling), MALAT1 also regulates tumor extrinsic pathways (osteoclast mediated bone resorption). Inhibiting MALAT1, or the miR-34a and miR-449 dependent derepression of c-Met and SOX4 that it produces, has been proposed as a way to limit progression and metastasis in osteosarcoma. If prognosis improves an intervention based on MALAT1 may be beneficial to patients with osteosarcoma and other bone cancer, which warrants further investigation; the molecular interactions remain unclear (2, 3).

A summary of the pathological role of MALAT1 dysregulation in major bone diseases, molecular mechanisms of MALAT1 regulation and therapeutic applications is summarized in Table 2. This review points out the clinical importance of MALAT1 as a biomarker and therapeutic target for bone diseases.

**Table 2:** Pathological significance of MALAT1 in bone-related diseases.

Disease	MALAT1 Expression Change	Key Molecular Mechanisms	Pathological Consequence	Therapeutic Potential	Key References	Evidence level and human validation
Osteoporosis	Downregulated in bone tissue and serum	β-catenin-OPG signaling ↓; miR-217/AKT3; miR-485-5p/WNT7B	Impaired osteogenesis; increased bone resorption; decreased BMD	Restoration of MALAT1; biomarker for diagnosis/monitoring	(14, 34, 45)	*In vitro* and rodent models, including ovariectomy and hindlimb unloading. Human evidence is observational only: MALAT1 expression in patient blood and bone. Association, not causation. No interventional study.
Osteoarthritis	Upregulated in cartilage and synovium	MALAT1 promotes MMP-13 and ADAMTS-5 via miR-145; JNK signaling	ECM degradation; cartilage damage	Targeting MALAT1 to suppress catabolic enzymes	(57, 58)	*In vitro* in rat and human chondrocytes. Human evidence is the correlation of MALAT1 with miR-145 and ADAMTS5 in patient cartilage. Association, not causation.
Osteosarcoma	Elevated in tumor cells	miR-34a and miR-449 sponging, derepressing c-Met and SOX4; Akt/mTOR and β-catenin pathways	Proliferation; invasion; metastasis; osteolysis	Anti-MALAT1 ASOs/siRNA; miR-34a restoration	(2, 59)	*In vitro* and cell line work; human evidence is tumour expression and its association with overall survival. One of the two citations is a narrative review rather than primary data. Association, not causation.
Multiple Myeloma	Elevated; m5C-modified exosomal MALAT1	NSUN2-mediated m5C methylation; RANKL/AKT/MAPK in osteoclast precursors	Osteoclast-mediated bone destruction	ASO gapmer targeting MALAT1; exosome-based therapy	(33, 60)	*In vitro* and mouse models. Human evidence is expression in patient plasma cells and clinicopathological association. Association, not causation.
Inflammatory Bone Diseases	Dysregulated in immune cells	NF-κB; miR-155/DC-SIGN/IL-10 axis; Th17/Treg balance	Enhanced osteoclastogenesis; pathological bone loss	Modulating MALAT1 in T cells/macrophages	(37, 38)	*In vitro* and mouse *in vivo*, in non-skeletal inflammatory models. Bone loss is inferred from the cytokine changes rather than measured.

The role of MALAT1 in bone related diseases. In the table below, an overview of molecular mechanisms, pathological consequences, and therapeutic potential of MALAT1 dysregulation in the main bone diseases (osteoporosis, osteoarthritis, osteosarcoma, multiple myeloma and inflammatory bone disorders) is shown. Aberrant MALAT1 expression is always associated with the disease progression, and this suggests that MALAT1 is a potential diagnostic/prognostic marker and therapeutic target. In this context, the regulatory activities of MALAT1 in osteogenesis, osteoclastogenesis, immunity and tumor biology bring to light its clinical value in several skeletal diseases.

## 9 The potential and challenges of MALAT1 as a therapeutic target for bone homeostasis

### 9.1 Design and optimization of MALAT1-targeted therapeutic strategies

Due to its important functions in regulating bone homeostasis and pathological conditions such as osteosarcoma, multiple myeloma and osteoporosis, the long non-coding RNA (lncRNA) MALAT1 is a subject of great interest for therapeutic strategies. In order to specifically target MALAT1 expression and activity, multiple strategies have been developed, targeting the rebalancing of bone and preventing the disease progression, which have been linked to the complex MALAT1 expression and activity regulation system.

Targeting of specific regions of MALAT1 by small interfering RNAs (siRNA) and antisense oligonucleotides (ASO) are the major approaches that are under consideration. These molecules have been demonstrated to regulate the expression of MALAT1 in various animal models, and to regulate bone remodeling processes. For instance, the downregulation of MALAT1 by siRNA in osteosarcoma cells inhibited growth, migration and invasion, thus suggesting that MALAT1 is a therapeutic target in bone cancers (59). Accordingly, antisense gapmer oligonucleotides against MALAT1 have been applied to models of multiple myeloma (MM), resulting in DNA damage and apoptosis and a decrease in the *in vivo* growth of MM (60). The results highlight the therapeutic potential of MALAT1 modulation using sequence specific oligonucleotides and their potential use in the treatment of bone related diseases.

A number of sophisticated nanocarrier systems have been created to enhance the efficacy and tissue specificity of therapeutics that target MALAT1. The delivery of siRNAs and ASOs to bone tissue via lipid-based nanoparticles and poly(lactic-co-glycolic acid) (PLGA) microparticles is a promising approach due to their targeted delivery and ability to overcome systemic delivery issues including rapid degradation and off-target effects. For example, fatty acid conjugation of antisense oligonucleotides has been shown to enhance plasma protein binding and consequently plasma levels and delivery to muscle tissue, which can be extended to systems for targeted delivery to bone tissue (61). These nanocarrier platforms show potential to enable the sustained delivery of MALAT1 inhibitors and facilitate their cellular uptake without the concurrent presence of high levels of MALAT1 inhibitors in the blood stream.

In recent years, efficient ways of genome-level modulation of MALAT1 expression have been developed, including genome editing technology, CRISPR-Cas9. Two things should be separated here. The loss-of-function work in cancer models that reduced metastasis and proliferation did not use CRISPR-Cas9: MALAT1 was disrupted by genomically integrating RNA-destabilising elements with zinc finger nucleases, and the therapeutic arm of that study used antisense oligonucleotides (1). CRISPR-based approaches to MALAT1 have so far been applied to pooled screening of long non-coding RNA localisation rather than as a therapeutic intervention (62). No CRISPR-mediated modulation of MALAT1 has been reported in any skeletal model, and clinical application to bone disease remains theoretical. Unlike transient knockdown, the silencing of MALAT1 mediated by CRISPR could offers long-lasting therapeutic effects by permanently silencing the MALAT1 transcript. The use of CRISPR-Cas9 may be extended to bone diseases like osteoporosis, as well as to bone defects to restore the normal bone homeostasis by correcting the MALAT1 mis-expression, but more *in vivo* studies are needed to make this a safe and effective approach.

In addition to molecule targeting using MALAT1 siRNAs, high end nanodelivery platforms for bone targeting and gene editing technologies for long-term modulation of the molecule will be designed and optimized. All of the above strategies are backed by extensive preclinical evidence of the role of MALAT1 in regulating osteoblast differentiation, osteosarcoma development and the development of multiple myeloma. Strategies for combinatorial therapy in the future would be improved by the ability to specifically target and efficiently deliver agents to modulate MALAT1 in bone diseases in a safe and effective manner that could be combined with gene editing using the CRISPR approach.

### 9.2 Challenges and future directions in MALAT1-targeted therapy

Although the function of lncRNA MALAT1 in bone homeostasis has yet to be translated clinically, there are many challenges to be addressed. One drawback is the wide tissue expression of MALAT1, which could pose a risk of off-target effects in the case of systemically targeted therapeutics. In addition to its involvement in bone remodeling, MALAT1 has been shown to have a crucial role in a wide range of physiological and pathological conditions, such as cancer progression, cardiovascular disorders and immune regulation (63–65). Developing bone targeting strategies is essential to avoid unwanted side-effects in other tissues since this is a widely used expression. A possible strategy is the use of promoters specific to bone, such that MALAT1-targeted intervention such as antisense oligonucleotides (ASOs) or RNA interference molecules would have a therapeutic effect only on bone cells. For instance, the systemic toxicity and therapeutic index of the drug can be optimized by using different bone marrow MSCs specific promoters and/or regulatory elements (14, 16).

A further challenge in developing therapeutics targeting MALAT1 is that it could be pro and anti bone. Under some circumstances, MALAT1 can promote osteoclastogenesis, while under other circumstances it can inhibit osteoclastic differentiation of the macrophages, and promote osteogenic differentiation of mesenchymal stem cells (MSCs) (14, 15, 32). This dual role poses a therapeutic challenge in designing therapeutics as the down- and upregulation of MALAT1 could upset the balance between bone formation and bone resorption. In osteoblasts, MALAT1 can sponge miR-96 and miR-93-5p, thus promoting osteogenic gene expression (e.g., Osterix, SMAD5) but simultaneously, it can target the miR-124/NFATc1 axis in bone marrow-derived macrophages, favoring osteoclastogenesis (15, 18, 22). To realize the therapeutic potential, it is crucial to achieve cell type specific and dynamic modulation of MALAT1 expression levels and activity, whilst preventing unwanted side effects. It could be through the development of delivery systems that could specifically target osteoblasts or osteoclast precursors, and/or the design of inducible expression systems that could control the expression of MALAT1 at specific times of bone remodeling.

The mechanism of spatiotemporal regulation of MALAT1 in the bone microenvironment is still unclear and requires further research. The fact that expression is in a defined pattern and that osteogenic and osteoclastic differentiation suggest context dependent action, means that its action is tightly controlled (14, 23). The expression pattern and the regulatory networks of MALAT1 can be dissectively studied at the cellular and tissue levels with unprecedented resolution using new technologies like single-cell RNA sequencing (scRNA-seq) and spatial transcriptomics. These strategies can be used to determine specific subpopulations of bone cells in which MALAT1 has key roles and can discover its interactions with other non-coding RNAs, transcription factors and signaling pathways, such as Wnt/β-catenin, Notch and OPG/Jagged1 axes (14, 64). The use of these data will facilitate the design of precision therapies with a more restricted activity in time and place, to improve the effectiveness and safety of MALAT1 targeting.

In addition, the development of innovative drug delivery systems is fundamental to bring the therapies to the clinic that are aimed at targeting MALAT1. In bone diseases, gold nanoparticle based systems coupled with antisense oligonucleotide have been also proved to be effective in reducing the expression of MALAT1, thus inhibiting the metastasis of cancer, suggesting their potential application in bone diseases (66). The ability to increase the delivery of such nanocarriers to bone, using ligands or peptides specific to the bone. In addition, the delivery of bone marrow mesenchymal stem cell (BMMSC)–derived MALAT1 or inhibitors of MALAT1 has been found to be effective in the repair of bones and regulation of osteoblast activity in osteoporotic models (16, 32). The potential for *in vivo* modulation of MALAT1 could be achieved through the application of the use of endogenous vesicle systems as biocompatible and efficient carrier systems.

The translational obstacles deserve to be stated more plainly than the preceding paragraphs do. MALAT1 is among the most abundant and most widely expressed transcripts in human tissue, and it is not a bone-restricted target; systemic suppression would act simultaneously on lung, liver, vasculature and immune compartments in which the same transcript has documented functions (8, 63). Because Malat1 loss in mice produces a skeletal phenotype without overt developmental abnormality, the consequences of long-term suppression in other organs cannot be inferred from those animals and would need to be measured directly. The modality itself carries known liabilities: phosphorothioate antisense oligonucleotides accumulate in liver and kidney, can cause dose-dependent thrombocytopenia and complement activation, and produce hybridisation dependent off-target cleavage of transcripts carrying partially complementary sequences, all of which are monitored in clinical antisense programmes (67). Delivery to bone is a further unsolved problem, since mineralised tissue is poorly perfused relative to liver and the osteoclast and osteoblast compartments must be reached selectively; bone-seeking strategies such as bisphosphonate or oligopeptide conjugation improve skeletal accumulation but have not been combined with MALAT1-directed oligonucleotides (68, 69). Finally, because the direction of the MALAT1 effect differs between osteoblast lineage and osteoclast precursor cells, an agent that is not cell-selective could worsen the imbalance it is intended to correct. None of these obstacles has been addressed for MALAT1 in a skeletal model, and they should be regarded as prerequisites rather than as refinements.

Finally, MALAT1 is a promising target for bone disorders because it fulfills multiple functions in both osteogenesis and osteoclastogenesis but targets are difficult due to its widespread expression and dual function. New challenges will have to be addressed in the future through new research opportunities such as state-of-the-art single cell and spatial RNA sequencing technology, new delivery platforms and new bone specific promoters. Targeting MALAT1 with these advances will enable the development of individual therapies that will bring high precision, efficacy and safety to the restoration of bone homeostasis and curing of bone disease.

## 10 Integrated network model of MALAT1 regulation in bone homeostasis and future research directions

### 10.1 Constructing a MALAT1-centered regulatory network model for bone homeostasis

A comprehensive regulatory network model of the long non-coding RNA (lncRNA) MALAT1 in the bone homeostasis is required to integrate multi-layered molecular information such as the signaling pathways, competing endogenous RNA (ceRNA) pathways, epigenetic regulation and modulation of the immune microenvironment. MALAT1 has been revealed to play a crucial role in bone metabolism, affecting the differentiation and activity of bone-forming osteoblasts, bone-resorbing osteoclasts, bone marrow resident cells such as mesenchymal stem cells (MSCs), and immune cells, thus controlling the complex regulation of bone remodeling.

The β-Catenin-OPG/Jagged1 axis, which is regulated by MALAT1, is one of the main signaling pathways of the network. The ability of MALAT1 to induce osteogenic differentiation is associated with its ability to up-regulate the expression of osteoprotegerin (OPG), a decoy receptor that has been demonstrated to inhibit osteoclastogenesis and Jagged1, a member of the Notch family that has been shown to be involved in the communication between osteoblasts and osteoclasts. For instance, MALAT1 was found to bind to miR-124-3p and facilitate the osteogenic differentiation of bone marrow-derived mesenchymal stem cells (BMSCs), which leads to the upregulation of IGF2BP1 and activation of the Wnt/β-catenin pathway, promoting increased osteogenesis and reduced osteoclastogenesis (14). Similarly, MALAT1 also regulates the expression of OPG and the proliferation of osteoblastic cells in human by regulating the signaling of RANKL, which plays a role in the regulation of the β-Catenin-OPG axis (20). This axis is an important regulatory point and MALAT1 is a key player in the regulation of bone formation and resorption.

In addition to direct signaling, MALAT1 could act as a ceRNA to competitively bind to microRNAs (miRNAs) and regulate downstream gene expression that is important for bone cell differentiation and function. For example, MALAT1 has been shown to bind to miR-124 to regulate its target gene NFATc1, which is a master regulator of osteoclastogenesis, and to accelerate differentiation of bone marrow-derived macrophages into osteoclasts (15). In osteoblasts and MSCs, MALAT1 influences the expression of transcription factors, SATB2 and Osterix (Osx), respectively, by binding to miR-34c and miR-96, both important regulators of osteoblast differentiation. Additionally, MALAT1 regulates the miR-93-5p/SMAD5 axis in periodontal ligament stem cells, and its expression changes in the osteogenic differentiation of periodontal ligament stem cells (22). These ceRNA interactions uncover a layer of post-transcriptional regulation that is known to regulate gene expression networks to promote either bone formation or bone resorption, depending on the cell type.

There's another part of the complicated role of MALAT1 in bone maintenance: the ability to control epigenetic processes. MALAT1 has been shown to associate to chromatin-modifying complexes to control the modification of histone proteins and transcription. For example, in hypertrophic cardiac remodeling, MALAT1 is able to bring in Suv39h1 to the MyoD-binding sites, leading to the trimethylation of H3K9 and transcriptional repression (70). This mechanism has been established in cardiovascular cells, and suggests that MALAT1 may be involved in epigenetic regulation of bone cells indirectly, by acting on key transcription factors that regulate bone formation, particularly Runx2 and Osterix. Actually, MALAT1 overcomes the negative regulation of miR-30 on Runx2 and therefore, epigenetic and post-transcriptional mechanisms can function together in the control of osteogenic gene expression (17). Epigenetic analysis of this information within the network model will help to understand the modulatory activity of MALAT1 on chromatin state that helps to maintain the commitment and function of bone cells.

The immune microenvironment is also important in bone remodeling and immune cells modulate osteoclastogenesis and osteoblast activity. Besides bone cells, MALAT1 also controls immune cells like macrophages, osteoclast precursors. In BMDMs, osteoblast-derived exosomes can induce osteoclastogenesis via MALAT1/miR-124/NFATc1 signaling axis, suggesting a bone-forming/bone-resorbing cell-to-cell communication by MALAT1 in BMDMs. Besides, MALAT1 has been shown to be involved in modulating inflammatory response by miRNA sponging, such as miR-150-5p in macrophages, which is related to cytokines production, and MALAT1 might be involved in bone resorption in inflammatory conditions (7). These findings highlight the importance of MALAT1 in controlling the immune microenvironment in bone, and its effects on immune cell activity and interactions with the bone.

The versatility of regulation of MALAT1 expression and activity is essential, with both spatial and temporal mechanisms being crucial. The expression pattern of MALAT1 is changing during the differentiation of osteoblasts, osteoclasts, MSCs and immune cells, as well as in response to changes in the environment (hypoxia and mechanical stress). During fracture healing, the miR-22-3p/CEBPD axis is crucial in driving osteogenesis that is regulated by hypoxia (47) in osteoblast precursor cells. Conversely, mechanical shear stress reduces MALAT1 levels in the MSCs and impedes the acquisition of an adipogenic phenotype while promoting an osteogenic phenotype, indicating that MALAT1 is mechanosensitive in regulating the differentiation of MSCs (42). The spatiotemporal regulatory activities underscore the importance of context-specific MALAT1 activity in bone homeostasis.

The remainder of this subsection is computational prediction rather than experimental finding, and is labelled as such. The network model forecasts connections between MALAT1 and other long non-coding RNAs and transcription factors that regulate bone cell function; none of the interactions named in this paragraph has been demonstrated experimentally in bone tissue. MALAT1 has the unique feature of being predicted to bind to multiple lncRNAs, such as H19 and MEG3, which are reported to regulate osteogenesis and bone metabolism. In addition, the H19 may affect the osteoblast differentiation process by regulating miRNAs and epigenetic modification, suggesting that H19 may have synergistic or antagonistic effects on the regulation network of MALAT1. The transcription factors Runx2 and NFATc1 are key components of this network that are regulated by MALAT1 through ceRNA and epigenetic mechanisms. These interactions are predicted and may provide a theoretical framework for experimental testing and for the dissection of the cooperative regulatory circuits of bone remodeling.

Finally, the molecular regulatory network related to the MALAT1 plays a complex role in bone homeostasis at multiple layers such as the β-Catenin-OPG/Jagged1 pathway, ceRNA-mediated miRNA sponging, epigenetic regulation and regulation of the immune microenvironment. The network explains the role of MALAT1 in the various bone remodelling processes (osteoblasts, osteoclasts, MSCs and immune cells) both in space and time. The interactions with other lncRNAs and the important transcription factors predicted further increase the complexity of the model, and provide a theoretical basis for future experiments on the modulation of MALAT1 for therapeutic use to modify bone disease.

### 10.2 Prospects of single-cell and spatial omics technologies in MALAT1 research

Single-cell RNA sequencing (scRNA-seq) and spatial transcriptomics allow for high-resolution measurement of gene expression, across the cell heterogeneity and spatial organization of complex tissues, a revolution in the study of gene expression. The technologies have great potential to further our understanding of the long non-coding RNA (lncRNA) MALAT1, especially in the context of bone homeostasis where cellular diversity and microenvironmental interactions are important. Single-cell RNA sequencing has not yet been applied to MALAT1 in the bone microenvironment. Were it applied to bone marrow stromal cells, osteocytes, osteoclasts and osteoblasts, it would separate the abundance of the transcript in each population from its abundance in whole bone, which bulk measurement cannot do. As MALAT1 has pleiotropic roles in gene expression, alternative splicing and cell signaling pathways, this technique may provide insights into the expression heterogeneity and functional specificity of MALAT1 in these cell types. In other systems, scRNA-seq uncovered the differential expression of MALAT1 with respect to both temporal and spatial dimensions, and it was proposed to exert a cell-type specific function in regulating cell proliferation, migration and differentiation, suggesting that similar regulation might be found in bone tissue (71, 72).

ST complements single-cell analysis by retaining spatial context of gene expression in the context of the tissue architecture. This technology can highlight the exact localization of MALAT1 transcripts in bone tissue, and can depict spatial distribution patterns across and through the bone tissue which will be correlated to bone remodeling units, vascular niches and other microdomains vital for bone homeostasis. This spatial information could be valuable to understanding the function of MALAT1 in the bone microenvironment, both for intercellular communication and signaling. For instance, the spatial transcriptomic profiling of the tumour microenvironment has identified the spatial association between the expression of MALAT1 and certain neighbourhoods and functional states, proposing locations for MALAT1 enriched microdomains in bone remodeling sites (71, 72). This spatial resolution also can explain the interaction between MALAT1 and major signaling pathways, such as β-Catenin-OPG/Jagged1 that play critical roles in the crosstalk of osteoblast and osteoclasts and bone remodeling.

The integration of the single-cell and spatiotemporal information can provide a fine-grained, spatiotemporal atlas of the expression and function of MALAT1 in bone tissue. This detailed map would allow for the identification of changes in the expression of MALAT1 over the course of bone development, remodeling and in disease states such as osteoporosis or fracture healing. Moreover, it could be applied to identify new, gene and signaling pathway networks of MALAT1 expression in specific cell types. Single cell and spatial resolution of MALAT1 role will also enable the precision therapeutic approaches for MALAT1. For instance, in the case of MALAT1, this can be accomplished by selectively inhibiting or modifying the expression of MALAT1 in osteoblasts and/or osteoclasts, while limiting other off-target activity and therapeutic efficacy (62, 72).

In addition, recent studies have shown the feasibility of imaging-based pooled CRISPR screening and single-cell transcriptomics to localize and function MALAT1 in the cell. These methods could be adapted to bone cells to dissect the molecular mechanisms involved in the nuclear localization of MALAT1, its association with chromatin modifiers, and its role in the regulation of gene expression programs known to be essential for bone cell differentiation and function that could be associated with the nuclear bodies like paraspeckles (62). This knowledge will help to elucidate the mechanism of action of MALAT1 in bone homeostasis.

Three specific questions raised earlier in this review are the ones these methods are positioned to settle. First, whether the abundance of MALAT1 reported in bone reflects enrichment in a particular cell population or uniform expression across the tissue, which bulk measurement cannot distinguish and which determines whether cell-selective targeting is feasible at all. Second, whether the opposing directions of effect described in Section 4.2 correspond to distinct cell states present at the same remodeling site, which would make the contradiction a sampling artefact rather than a biological one. Third, whether the ceRNA interactions in Section 4 occur at transcript ratios that make competition plausible, since single-molecule and single-cell quantification measures the abundances that the sponge model requires and that bulk assays obscure.

### 10.3 Key pathways from basic research to clinical translation

#### 10.3.1 Biomarker validation in large clinical cohorts

A molecular regulator found in basic studies must be properly tested in large clinical cohorts to find out if it can be used as a clinically useful biologic marker for bone diseases. *In vitro* and animal studies have shown that MALAT1 regulates the osteogenic differentiation, osteoclastogenesis and bone remodeling by various signaling pathways, including miR-124/NFATc1, miR-96/Osterix and miR-124-3p/IGF2BP1/Wnt/β-catenin (14, 15, 18). The mechanism, however, must be confirmed in humans to decide on the clinical significance of MALAT1. For instance, MALAT1 is linked with the osteogenic activity of bone marrow stem cells and the osteoclast precursors, which suggest that MALAT1 can be used to ascertain the rate of osteoporosis and other metabolic bone illness (14, 18). Although these are encouraging results, the diversity of bone diseases as well as the complexity of bone remodeling calls for the availability of large well-characterized patient cohorts in order to evaluate the sensitivity, specificity and the prognosis of MALAT1. These studies should encompass a diverse population of people that have different amounts of bone density loss, fracture risk, and disease course. Moreover, longitudinal cohort studies would enable the investigation of the changes of MALAT1 expression in the course of the disease, and the study of MALAT1 expression in response to treatment. MALAT1 expression data could also be integrated with other established clinical parameters including bone mineral density (BMD), bone turnover markers and imaging parameters to further reveal its potential to be a diagnostic and prognostic biomarker. Moreover, an ability to detect MALAT1 in easily obtainable biological material like peripheral blood leukocytes (PBLs) or bone marrow aspirates would be supporting the clinical applications. If MALAT1 is confirmed as a reliable biomarker, it could contribute to earlier diagnosis and to individualised treatment of bone diseases. That confirmation has not yet been obtained, and the studies required to obtain it are set out below.

#### 10.3.2 Bone-targeted delivery systems and preclinical safety

The mechanism by which MALAT1 based therapeutics could be delivered safely and effectively to the bone to bring these therapeutics from bench to bedside. Since MALAT1 is associated with the regulation of osteoblast and osteoclast differentiation, modulation of MALAT1 in the bone could be a potential therapeutic approach in bone diseases such as osteoporosis, osteoarthritis and bone defects (14, 22). Systemic delivery of MALAT1 systemic therapy, however, faces obstacles of off-target effects, fast degradation and low specificity. To resolve these problems, bone-targeted delivery systems such as nanoparticles, exosomes and conjugated oligonucleotides have been studied. This may include the potential of the exosomes, which are derived from bone marrow mesenchymal stem cells (BMSCs) to carry MALAT1 and to promote osteoblast activity (16). Moreover, antisense oligonucleotides (ASOs) conjugated with the peptide that targets the nucleus have been found to be effective in reducing expression of MALAT1 in cancer cells, and this could be a source of inspiration for some of the strategies used in bone targeted applications (66). To maximize the efficacy with minimum systemic exposure, bone affinity, cellular uptake by osteoblastic and osteoclastic cells, and control over the release kinetics of the delivery system should be maximized. A large number of the delivery systems need to be evaluated in relevant animal models for biodistribution, pharmacokinetics and immunogenicity and possible toxicity. Long-term studies are particularly useful for the evaluation of long-term efficacy in order to promote long-term bone regeneration or pathological bone resorption or for the monitoring of side effects, such as immune response or off-target gene modulation. Furthermore, animal models which recapitulate human disease pathology such as osteoporosis and fracture healing models are excellent platforms for testing MALAT1-based therapeutics (32, 47). If these bone-targeted MALAT1 delivery systems can be developed and validated, they will provide new and exciting ways to treat bone disorders in a more precise way, and eventually lead to clinical trials.

#### 10.3.3 Multicentre clinical trials and outcome measures

Appropriate multicenter clinical trials are crucial to bring MALAT1 targeted interventions to the clinical practice. The therapeutic efficacy and safety of modulation of MALAT1 in patients with prevalent bone diseases (including osteoporosis, osteoarthritis and bone defects to be repaired) should be evaluated during these trials. Targeting MALAT1 can be a promising approach to restore bone homeostasis since MALAT1 is involved in many molecular pathways in osteogenic differentiation, osteoclastogenesis, and bone remodeling, such as the miR-93-5p/SMAD5 axis in periodontal ligament stem cells and the miR-124/NFATc1 axis in osteoclast precursors (15, 22). Comprehensive outcome measures need to be used in clinical trials to capture multifactorial aspects of bone health. These include quantitative measurements of bone mineral density (BMD) by dual energy x-ray absorptiometry (DXA), bone microarchitecture by high-resolution peripheral quantitative computed tomography (HR-pQCT) and biochemical markers of bone formation and resorption such as serum bone alkaline phosphatase, serum osteocalcin and C terminal telopeptide (CTX). Functional outcomes of fracture risk reduction, pain relief and increased mobility should also be thought of within the realm of clinical benefit. The expression of MALAT1 in the patient at the time of diagnosis and/or the genetic variants of MALAT1 can be used to stratify patients, which can enable individualized therapeutic approaches. Furthermore, toxicity involving other organ systems (immune response, etc.) should be assessed as MALAT1 is involved in various physiological and pathological processes like cancer and cardiovascular diseases (4, 65). Multicenter collaboration will help recruit a diverse patient population, increase statistical power and results will be generalizable. Overall, these clinical trials will help to generate the data needed to incorporate MALAT1-targeted treatments into routine treatment for bone diseases, leading to better outcomes and quality of life for patients.

## 11 Limitations of this review and literature search strategy

This is a narrative rather than a systematic review, and its limitations follow from that design. Records were identified in PubMed and Web of Science from database inception to September 2026 using combinations of the terms MALAT1, NEAT2, long non-coding RNA, bone homeostasis, bone remodeling, osteoblast, osteoclast, osteoporosis, osteoarthritis, osteosarcoma and mesenchymal stem cell, supplemented by screening the reference lists of the retrieved articles. Selection was made by the authors on the basis of relevance to MALAT1-dependent mechanisms in skeletal tissue; no predefined protocol, no duplicate independent screening and no formal risk-of-bias assessment were applied, and no quantitative synthesis was attempted. Only English-language and Chinese-language records were considered. Three further limitations apply to the evidence itself rather than to the search. Most of the primary studies use transient overexpression or knockdown in cell lines, a design that is prone to publication bias towards positive and directionally clean results. Effects reported in non-skeletal systems are included where they identify a molecular step, but these cannot be assumed to transfer to bone. Finally, the number of independent *in vivo* studies is small, and much of the mechanistic framework in Section 10 rests on a single laboratory's mouse work, so the integrated network model presented there should be treated as a hypothesis for testing rather than as an established description.

## 12 Conclusion

The knowledge of the different roles of MALAT1 in bone biology has taken a giant leap forward in the comprehension of bone homeostasis and pathologies. The multifaceted regulation by MALAT1 in the β-Catenin-OPG/Jagged1 axis, the ceRNA mechanism and epigenetic regulation all underscore the important role of MALAT1 in regulating the balance between the differentiation and activity of osteoblasts and osteoclasts. This is a two way system which is necessary for balance in bone remodeling (needed for bone integrity and function). The challenge of the complexity of bone cell biology is illustrated by the complexity of the mechanism of MALAT1 action and the complexity of the molecular pathways, and the need for multiple research results to be integrated in order that an overall understanding of the role of MALAT1 can be gained.

Moreover, MALAT1 is not only involved in bone microenvironment regulation but also in the fate of mesenchymal stem cells as well as in the regulation of immune cells. The concept of bone homeostasis is extended to a complex regulatory network regulated by MALAT1 that takes into account the fine-tuned interaction between the cells that comprise the bone niche. The findings describe the bone microenvironment as a complex system in which MALAT1 acts across several cell types and signaling pathways. Translating these together, in a holistic perspective, will increase our understanding of skeletal biology, integrating the molecular signaling, cellular differentiation and interactions with the microenvironment.

In pathological situations, however, like osteoporosis, osteoarthritis, and bone tumors, MALAT1 expression is associated with the progression of the disease, and this gene could be utilized as a biomarker for the disease and be a therapeutic target. Because of its dual role in disease, the function of MALAT1 is complex and can be causative in disease-associated pathological remodeling, and can also be a compensation mechanism in a cell specific context and disease stages. A balanced approach is required to interpret the experimental data and clinical observations, and this should include a combination of molecular data with the results of the test. This approach will play a vital role in the clinical implementation and application of MALAT1, to ensure efficacy and applicability of the treatment in the context of disease.

In the future, the spatiotemporal specificity of the regulation of MALAT1 in bone tissues should be studied. Single cell and spatial omics technologies, at a high level of functional heterogeneity, have opened new opportunities to analyze MALAT1. These cutting edge techniques will enable researchers to identify the activity of MALAT1 in different types of cells and in different microenvironmental niches and discover new roles for MALAT1 that had not been found in bulk analysis. This detailed knowledge is crucial to developing valid interventions that will selectively inhibit the pathological functions of MALAT1, without affecting its physiological functions.

In addition, the development of bone-specific targeted delivery is a key translational challenge. Selective and effective delivery of modulators to bone will enhance therapeutic efficacy, but reduce off-target activities that have hindered the translation of RNA therapeutics to the clinic. These challenges and the introduction of MALAT1-targeted therapeutics into the clinic require an improved understanding of the molecular biology, bioengineering and pharmacology of these molecules.

Overall, the studies on MALAT1 in bone biology illustrate the intricacy and potential of investigating ncRNAs as part of a multifactorial system. The potential for MALAT1 as a biomarker and therapeutic target will require continued interdisciplinary efforts and the use of cutting-edge technologies and innovative strategies for delivering the work. These advances could change the way bone diseases are treated, and enhance the results of precision medicine strategies to treat bone disease.
